# Mitochondrial and NAD^+^ metabolism predict recovery from acute kidney injury in a diverse mouse population

**DOI:** 10.1172/jci.insight.164626

**Published:** 2023-02-08

**Authors:** Jean-David Morel, Maroun Bou Sleiman, Terytty Yang Li, Giacomo von Alvensleben, Alexis M. Bachmann, Dina Hofer, Ellen Broeckx, Jing Ying Ma, Vinicius Carreira, Tao Chen, Nabil Azhar, Romer A. Gonzalez-Villalobos, Matthew Breyer, Dermot Reilly, Shannon Mullican, Johan Auwerx

**Affiliations:** 1 Laboratory of Integrative Systems Physiology, Institute of Bioengineering, Ecole Polytechnique Fédérale de Lausanne (EPFL), Lausanne, Switzerland.; 2 Janssen Research and Development LLC, Raritan, New Jersey, USA.

**Keywords:** Nephrology, Bioinformatics, Chronic kidney disease

## Abstract

Acute kidney failure and chronic kidney disease are global health issues steadily rising in incidence and prevalence. Animal models on a single genetic background have so far failed to recapitulate the clinical presentation of human nephropathies. Here, we used a simple model of folic acid–induced kidney injury in 7 highly diverse mouse strains. We measured plasma and urine parameters, as well as renal histopathology and mRNA expression data, at 1, 2, and 6 weeks after injury, covering the early recovery and long-term remission. We observed an extensive strain-specific response ranging from complete resistance of the CAST/EiJ to high sensitivity of the C57BL/6J, DBA/2J, and PWK/PhJ strains. In susceptible strains, the severe early kidney injury was accompanied by the induction of mitochondrial stress response (MSR) genes and the attenuation of NAD^+^ synthesis pathways. This is associated with delayed healing and a prolonged inflammatory and adaptive immune response 6 weeks after insult, heralding a transition to chronic kidney disease. Through a thorough comparison of the transcriptomic response in mouse and human disease, we show that critical metabolic gene alterations were shared across species, and we highlight the PWK/PhJ strain as an emergent model of transition from acute kidney injury to chronic disease.

## Introduction

Kidney disease is a global burden on our health system. A large fraction of the population — more than 20% for acute kidney injury (AKI) (1) and for 10% for chronic kidney disease (CKD) (2) — suffer from kidney diseases. Despite being largely preventable, kidney disease is both a direct cause of morbidity and mortality as well as an important risk factor for various cardiovascular diseases (1). AKI is mainly triggered by environmental factors — hypovolemic shock (e.g., infection, bleeding, vascular surgery), heart failure, and exposure to nephrotoxins (e.g., antibiotics, chemotherapy, contrast agents) (3). Delayed graft failure, which is common in deceased donor transplants, is also a form of AKI of the transplanted kidney (3). Although AKI can be reversible, a portion of AKI patients, especially those suffering from repeated injuries, do not recover fully and eventually develop CKD (1). The severity of AKI can be influenced by genetics, from highly penetrant monogenic diseases to complex and multifactorial polygenic diseases (4, 5). Likewise in animal models, different genetic backgrounds affect the onset and progression of kidney diseases induced either by a single-gene mutation (6) or the response to toxins (7). The mechanisms and genetic determinants underpinning the severity of AKI and whether subjects recover from AKI or progress to CKD are poorly understood (8). There exists no treatment for AKI, to date, and a mechanistic understanding of the disease and how it can progress to kidney fibrosis and CKD are urgently needed to formulate therapeutic strategies.

**Figure 1:**
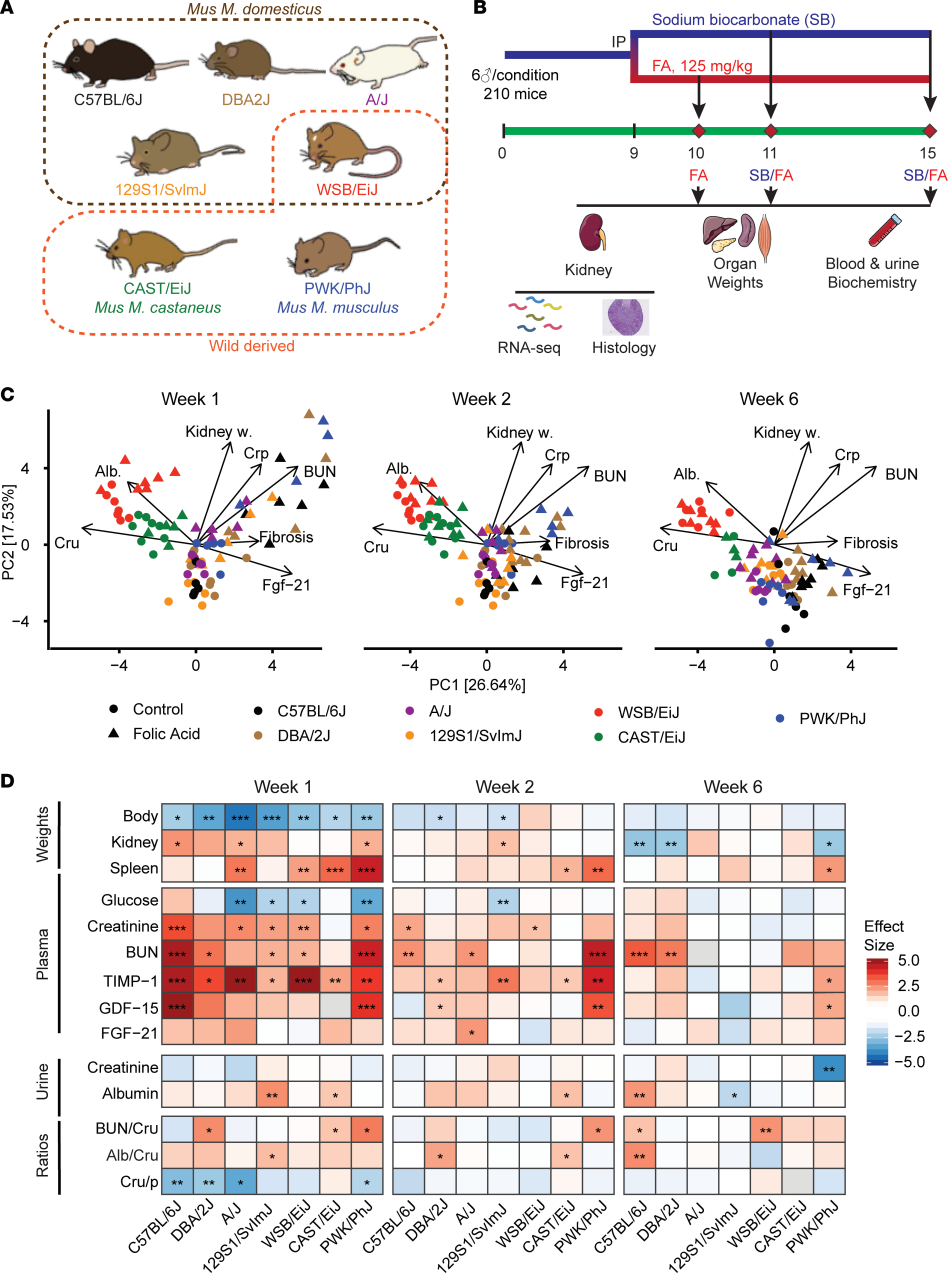
Strain-dependent responses to folic acid–induced kidney injury and their kinetics. (**A**) The 7 selected mouse strains of the study, indicating the wild-derived strains (WSB/EiJ, CAST/EiJ, and PWK/PhJ) and the 2 strains from a different subspecies from *Mus*. *musculus domesticus*. Strain images are from ref. 9. (**B**) Experimental design of the study. Six male animals at 8 weeks of age were used per strain and condition. There were 5 experimental conditions: control (sodium bicarbonate) weeks 2 and 6, and folic acid weeks 1, 2, and 6 after injection. The total number of mice was 210, although only 202 reached the end of the experiment. At the end of the experiment, none of the conditions had fewer than 4 mice. (**C**) Principal component analysis of all measured phenotypes across all time points. The PCA is represented 3 times to highlight the time evolution of phenotypes. Loadings of selected kidney-related phenotypes are shown. (**D**) Comparison of the folic acid–induced effects on the plasma, urine, and morphological traits across strains and time points. Two-tailed Student *t* test *P* values were adjusted for multiple testing by Benjamin-Hochberg method (***P < 0.001, **P < 0.01, *P < 0.05). Cr_p_, plasma creatinine; Cr_u_, urine creatinine; Alb, urine albumin; BUN, blood urea nitrogen; SR, Sirius red staining quantification; TIMP-1, Tissue Inhibitor of Metalloproteases 1; GDF-15, Growth/Differentiation Factor-15; FGF-21, fibroblast growth factor 2; BUN/Cr_u_, urea nitrog/creatinine ratio; Alb/Cr_u_, urine albumin/creatinine ratio; Cr_u/p_, Creatinine urine/plasma ratio. Values are Cohen’s effect sizes.

In this study, we characterized the response to kidney injury in 7 mouse inbred strains known as the founders of the Collaborative Cross (CC), Diversity Outbred (DO), and BXD populations (9) (Figure 1A).The DO population has previously been used to investigate the genetic causes behind variations in glomerular filtration rate (GFR) assessed through Cystatin C (10), and researchers found associations with type I IFN genes and inflammatory pathways, but there was no induction of overt kidney disease. In a cross between X-Linked Alport Syndrome mice and DO mice, authors found associations between the X-linked gene *Fmn1*, albuminuria, and GFR disruption (11). The purpose of our study was to establish a baseline of the responses of the founder strains of the DO population to AKI, to later define a suitable genetic cross to find the underlying genetic cause of susceptibility to AKI and transition to CKD.

Rodent models of AKI consisting of various chemical (e.g., cisplatin, folic acid [FA]) or surgical (ischemia reperfusion injury [IRI] and colon ligation puncture [CLP]) injuries that impact on the kidney (12) often fail to reproduce the full features of human diseases (13). Among AKI models, FA-induced nephropathy is a good compromise between simplicity and relevance to human kidney diseases (14), and it is frequently used in preclinical drug development (15, 16). In rodents, yellow crystals of FA are detected in the kidney as early as 30 minutes after injection with a toxic dose of FA (440 mg/kg); then, those crystals are fully cleared over approximately 4 days (14). Such high concentrations of FA lead to kidney injury, both through crystal toxicity and direct nephrotoxicity (17, 18), causing an increase of serum creatinine, blood urea nitrogen (BUN), and urine glucose levels (14).

In this study, we followed the evolution of injury and remission after the expected clearance of crystals 1, 2, and 6 weeks after a i.p. FA injection in 5 domesticated (C57BL/6J, DBA/2J, A/J, 129S1/SvlmJ, WSB/EiJ) and 2 wild-derived (CAST/EiJ, PWK/PhJ) inbred mouse strains (Figure 1A). After a short pilot experiment, we chose a mild (125 mg/kg) dose of FA because higher doses caused severe toxicity, leading to death of some strains. We measured plasma and urine biomarkers, as well as histological kidney features, and determined the renal transcript profiles. Through this approach, we determined that the C57BL/6J, DBA/2J, and PWK/PhJ mouse strains are the most susceptible strains to AKI and possibly to the transition to CKD. In addition, we observed that modulation of immune and mitochondrial pathways, notably NAD^+^ metabolism, during the initial recovery are predictive of long-term remission. We provide access to all data in this study through public repositories, as well as an interactive app, enabling users to explore and analyze all traits and gene expression changes observed in this study at www.systems-genetics.org/CC_founders_AKI This resource will help guide the selection of relevant strains for specific kidney disease modeling, thereby improving the translational potential of further research.

## Results

### Mouse strains exhibit a wide range of responses to AKI, from full resistance to chronic disease.

Kidney injury was induced by injecting a single dose of folic acid (FA) i.p. (125 mg/kg in 0.3M of sodium bicarbonate) in 8-week-old male mice (*n* = 6 per strain and condition for a total of 210 animals). Mice were sacrificed 1, 2, or 6 weeks after dosing, in order to capture the initial recovery and long-term remission phases (Figure 1B). In accordance with the 3R principles (replace, reduce, refine), control mice were only sacrificed at 2 or 6 weeks. Therefore, throughout the manuscript, week 1 FA-treated mice were always compared with week 2 control mice.

We performed principal component analysis (PCA) on the phenotype data of all mice to explore the relationship between strains and conditions as a function of time (Figure 1C). Control mice of the wild-derived WSB/EiJ and CAST/EiJ are separated from the other strains on the first component, which explains 26.64% of the variance. The WSB/EiJ — and, to a lesser extent, CAST/EiJ — have high baseline urinary albumin and creatinine and low FGF-21 (Figure 1C, online app; www.systems-genetics.org/CC_founders_AKI). After 1 week of FA injection, the measured phenomes of PWK/PhJ, DBA/2J, and C57BL/6J mice shift toward the upper right quadrant of the PCA plot. This shift is associated with an increase in plasma BUN and creatinine, kidney weight, and fibrosis measured by quantification of Sirius red staining in kidney sections. At weeks 2 and 6, the FA mice from all strains gradually clustered back with the control mice, except for PWK/PhJ, C57BL/6J, and DBA/2J in the lower right quadrant characterized by high circulating Fgf-21 and kidney fibrosis (Figure 1C, online app), suggestive of a slow recovery or persistent disease at week 6.

Body weight (BW) was measured throughout the study as a general readout of disease severity and its subsequent recovery. It decreased after 1 week of FA in most strains (Figure 1D). The A/J and 129S1/SvlmJ strains were most prone to BW loss, while the wild-derived strain CAST/EiJ lost almost no weight upon FA injury. Consistently, food intake decreased in laboratory mouse strains but not in wild-derived mice (Supplemental Figure 1A; supplemental material available online with this article; https://doi.org/10.1172/jci.insight.164626DS1). All strains recovered normal BW at week 2, except for the DBA/2J and 129S1/SvlmJ strains (Figure 1D). Kidney weight increased in the acute phase in some responsive strains (C57BL/6J, A/J, and PWK/PhJ; Figure 1D), as previously reported (14); then, it was reduced below the initial weight during the recovery phase at 6 weeks (Figure 1D). Spleen size increased by 40% in the PWK/PhJ strain, reflecting immune activation, and this remained the case even after 6 weeks, perhaps suggesting the onset of chronic inflammation. Other strains (CAST/EiJ, A/J, and WSB/EiJ) transiently gained spleen weight at week 1, but this was resolved before week 6 (Figure 1D and Supplemental Figure 1B). We observed no major weight or visual aspect changes in other organs (Supplemental Figure 1B).

Plasma and urine parameters were collected to assess the severity of kidney injury (Figure 1D and Supplemental Figure 2). Glycemia decreased upon FA in the strains that had the greatest change in BW (A/J, 129S1/SvlmJ, WSB/EiJ, and PWK/PhJ) and was maintained only in CAST/EiJ. Plasma markers of kidney injury — namely creatinine, BUN, and TIMP-1 — increased in all strains except for the highly resistant CAST/EiJ that only showed a transient increase in TIMP-1. While creatinine and TIMP-1 resolved itself in most strains, BUN remained high in 2 strains even at 6 weeks.

Urine creatinine and albumin levels, and the respective ratios, were less responsive than plasma levels (Figure 1D and Supplemental Figure 2), possibly because there was a difference in urinary output, which we unfortunately did not measure and could partially compensate for high urinary protein (19). Urine creatinine levels were only significantly affected by FA in PWK/PhJ (week 6). Urine albumin levels were affected in 129S1/SvlmJ (increased at week 1), CAST/EiJ (increased at week 1 and week 2), and C57BL/6J (increased at week 6). In addition, the urine albumin/creatinine (Alb/Cr_u_) ratio indicated that 129S1/SvlmJ (week 1), DBA/2J, CAST/EiJ (week 2), and C57BL/6J (week 6) are the most sensitive. The creatinine urine/plasma (Cr_u/p_) ratio seems to be consistent with the plasma parameters and reflects a large decrease in C57BL/6J, DBA/2J, A/J, and PWK/PhJ.

The circulating factors GDF-15 and FGF-21 are known to have a prognostic and even predictive value in CKD in humans (20, 21), but these markers are not exclusive to kidney injuries and are elevated in a wide range of conditions involving mitochondrial stress and injury, giving them the designation of mitokines (22). Both mitokines were elevated in most strains at week 1 (Figure 1D), but there was some heterogeneity between strains, with the C57BL/6J and PWK/PhJ strains showing a far stronger induction of GDF-15, the same strains that retain elevated kidney injury markers at week 6. This result may indicate that mitochondrial stress is a predictor of the susceptibility to AKI.

Overall, these results allow us to classify the strains into 3 groups, based on their response to FA-mediated kidney injury: Group I indicates resistant strains, including CAST/EiJ, a wild-derived strain from the *Mus musculus castaneus* subspecies, which shows only a minimal response to injury. Group II indicates recovering strains (A/J, 129SvlmJ, WSB/EiJ). These strains show a variable initial response to injury but subsequently recover almost fully at 6 weeks. Group III indicates sensitive strains (C57BL/6J, PWK/PhJ, and — to an extent — DBA2/J). These strains retain strong markers of kidney injury and/or inflammation at 6 weeks, indicating a very slow recovery or a transition to chronic disease.

### Kidney histopathology recapitulates strain-specific responses to kidney injury.

**Figure 2:**
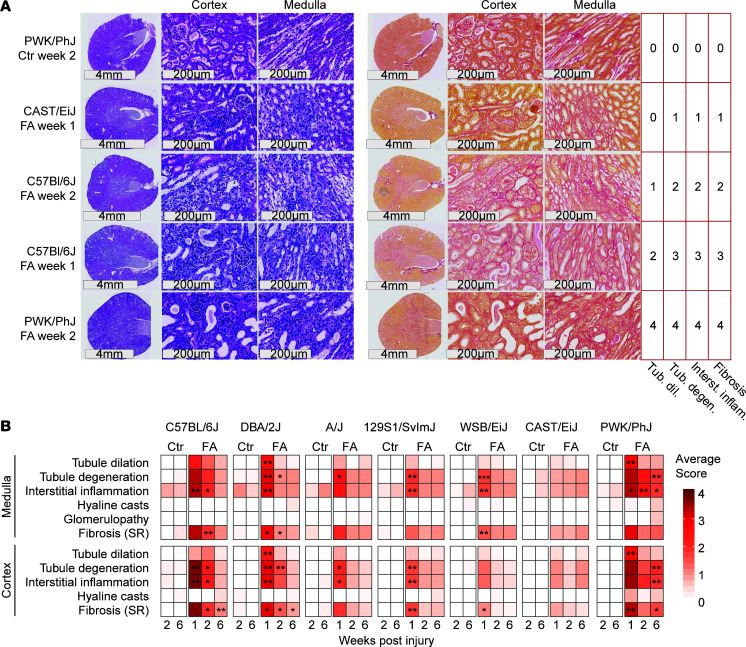
Strain-specific histological changes in the kidney upon folic acid injury. Histology slides were scored by pathologist in a double-blind manner. (**A**) Representative images of severity grade 1–4 kidney tubule dilation, tubule degeneration, interstitial inflammation, and fibrosis. Strains and time points are indicated on the left, and severity grades are indicated on the right. (**B**) Heatmap of the severity grades of kidney histology in each strain and time point. Statistical significance is shown representing Wilcoxon test comparison between FA and control, using FDR-corrected *P* values (***P < 0.001, **P < 0.01,*P < 0.05).

We performed H&E and Sirius red staining of parallel kidney sections of each animal and focused on the same region of the cortex and medulla in each staining (Figure 2A). The medulla and cortex were scored by a pathologist in a double-blinded manner. Representative examples of images corresponding to a severity from 0 to 4 are shown with the corresponding score (Figure 2A), along with the full quantification (Figure 2B).

The histological severity scores in both medulla and cortex closely followed the trends that were observed in the biochemical parameters of kidney injury. The CAST/EiJ (Group I) strain showed no significant difference with controls at any time, suggesting resistance to injury. Group II strains (A/J, 129SvlmJ, WSB/EiJ) displayed mostly tubule degeneration, interstitial inflammation, and fibrosis but no tubule dilation at week 1 and had no significant histological markers from week 2 onwards, indicating a fast recovery from injury (Figure 2B). At week 1, sensitive strains (Group III: C57BL/6J, PWK/PhJ, DBA2/J) showed tubule dilation, tubule degeneration, interstitial inflammation, and fibrosis (Figure 2B); although variability was high and there were high average scores, these data did not always reach statistical significance. Group III strains retained tubule degeneration and inflammation (PWK/PhJ and DBA/2J) or fibrosis (C57BL/6J and PWK/PhJ) even after 6 weeks (Figure 2B). Notably, we detected no hyaline casts or glomerulopathy in any of the strains — changes that are not expected in AKI and help explain the mild changes observed in the Alb/Cr_u_ ratio.

### The kidney transcriptomic response to AKI is qualitatively similar across strains.

**Figure 3:**
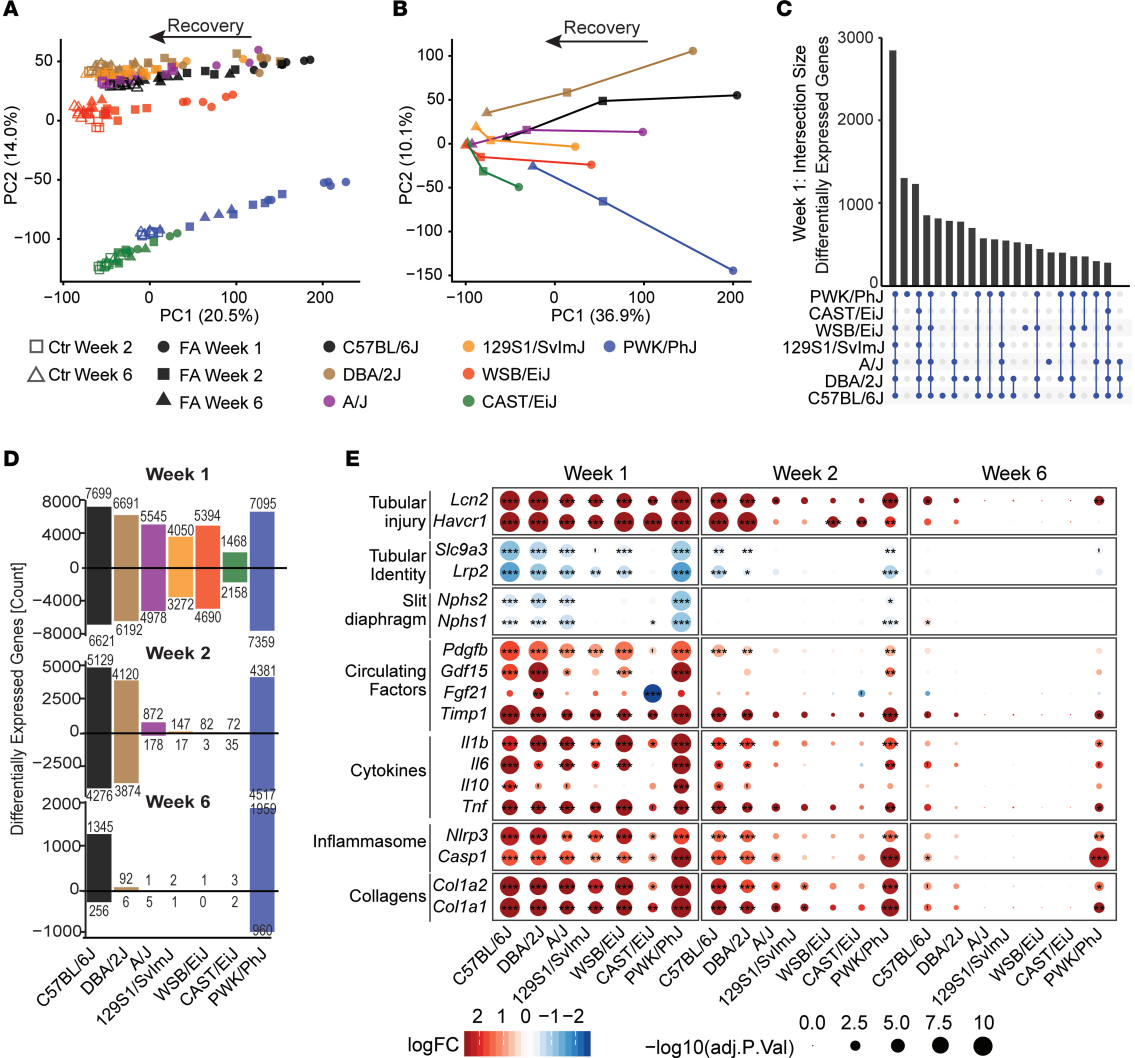
Kidney transcriptomic response to folic-acid-induced injury. (**A**) Principal component analysis of transcript data categorized by strain, time points, and control conditions. The first principal component associates with the severity of the injury, and its recovery, while the second principal component differentiates the strains, with the CAST/EiJ (*Mus musculus castaneus*) and PWK/PhJ (*Mus musculus musculus*) being most strongly separated from the other Mus musculus domesticus strains. (**B**) Principal component analysis of the fold changes (folic acid versus control) in all strains and time points. At week 1 (circles), strains can be easily divided between light (CAST/EiJ, 129S1/SvlmJ, WSB/EiJ) responses and more severe ones (PWK/PhJ, C57BL/6J, DBA/2J). Conversely, at 6 weeks (triangles), strains divide between nearly full recovery (most strains) and incomplete recovery (PWK/PhJ, C57BL/6J). (**C**) Upset plot presenting the number of overlapping differentially expressed genes in the same direction (treatment versus control) across strains at week 1. (**D**) Number of upregulated or downregulated genes per strain and time point. (**E**) Selected renal transcript markers of the severity of tissue injury and inflammation. The initial transcriptomic response at week 1 is similar across all strains, although the intensity varies, being smallest in CAST/EiJ mice. It comprises markers of tubular injury, loss of tubular and slit diaphragm identity, increase of circulating factors indicating mitochondrial strains as well as both pro- (*Il6*, *Il-1b*, *Tnf*) and antiinflammatory (*Il10*) cytokines. The components of the inflammasome are induced, as well as collagens (here Col1a1 and Col1a2 are shown, but many more collagens are induced). Notably, this transcriptomic response is maintained until 6-weeks post injection only on in the PWK/PhJ and C57BL/6J strains, indicating a potential transition to chronic disease. FDR-corrected *P* values; **P < 0.01, *P < 0.05.

To gain insight into the molecular pathways associated with the strain differences in kidney injury severity, we profiled the kidney transcriptomes of all 202 mice using RNA-Seq analysis. Through PCAs of the expression levels (Figure 3A) and the fold changes compared with control (Figure 3B), we observed that the principal component 1 (PC1) separated strains according to treatment and time, while the second PC2 separated strains from each other. PC2 is driven by mouse strain differences, which are greatest in different subspecies’ PWK/PhJ (*Mus musculus musculus*) and CAST/EiJ (*Mus musculus castaneus*) (Figure 1A).

Despite the differences in injury severity and recovery between strains, their transcriptomic response at week 1 was remarkably similar. At week 1, approximately 1,300 genes were differentially expressed in the same direction across all 7 strains (Figure 3C). A further ~2,800 genes were shared by all strains but the resistant CAST/EiJ. This similarity ended at weeks 2 and 6 when several strains no longer had any significant differentially expressed genes (Figure 3D), showing that all strains but the resistant CAST/EiJ reacted similarly to injury but that their responses diverged in the subsequent remission phase.

### The intensity of the kidney transcriptomic response parallels the severity of clinical injury.

To better understand the transcriptomic response to FA, we performed a PCA of the expression fold changes between treatment and controls of the different strains at different time points (Figure 3B). Interestingly, the first PC capturing 27% of variation faithfully traces the trajectory of the strains as they react to the injury and recover from it to varying extents, mirroring the histological, biochemical, and clinical phenotypes (Figure 3B). C57BL/6J, DBA/2J, A/J, and PWK/PhJ are the most responsive strains related to the transcripts that are consistently changed in the population. 129S1/SvlmJ and WSB/EiJ were less responsive, whereas CAST/EiJ was again resistant to changes in transcript levels induced by FA kidney injury. The sensitive strains in Group III (C57BL/6J and PWK/PhJ) retained a strong transcriptomic injury footprint at week 6. It is worth noting that the transcriptional effect of FA on PWK/PhJ at week 6 (Figure 3, B and D) was larger in magnitude than the acute effect on the CAST/EiJ mice, showing again the extensive range of severity in these strains.

The overall transcriptomic response was mirrored by an increase in markers of tubular and kidney injury (*Lcn2*, *Hacr1*, *Pdgfb*; Figure 3E) and a decrease in markers of tubular identity (*Slc9a3* and *Lrp2*) and slit diaphragm of podocytes (*Nphs1*, *Nphs2*), a critical component of the glomerular filter of the kidney. The circulating markers observed in the plasma (*Gdf-15*, *Fgf*-*21*, and *Timp1*) were also expressed higher at the transcript level, confirming their renal origin. Expression of transcripts encoding for different collagens (*Col1a1*, *Col1a2*, and many others) was higher across all strains, indicating fibrogenesis, while inflammatory (*Il1b*, *Il6*, *Tnf*) and antiinflammatory (*Il10*) cytokines increased together with the main components of the inflammasome, *Nlrp3*, and *Casp1*.

The resistant CAST/EiJ strain showed increased transcript levels encoding for kidney injury markers *Lcn2* and *Havcr1* (Figure 3E) in the wake of mildly induced inflammatory mediators and collagens, indicating that it does suffer an initial injury. However, tubular and slit diaphragm proteins and, notably, the hallmarks of mitochondrial stress, *Fgf21* and *Gdf15*, were not induced, and *Fgf21* was significantly reduced (Figure 3E). Conversely in the Group III strains, PWK/PhJ, DBA/2J, and C57BL/6J, most markers of kidney injury and identity were normalized at week 6; however, these sensitive strains retained a robust expression of proinflammatory cytokines, inflammasome components, and collagens, indicating a transition from acute injury to chronic inflammation and fibrosis. Deficiency in EGF and TGF-α signaling through EGFR has been shown to be an important modifier of kidney structure and function and a risk factor in renal diseases (23). The baseline expression of *Egf* was near constant across strains but declined strongly in all strains except the CAST/EiJ upon injury (Supplemental Figure 3A). Conversely, *Tgfa* expression was lower and varied across strains at baseline but did not correlate with injury (Supplemental Figure 3A). Despite these changes, the downstream targets of the pathway were not significantly enriched upon injury in any of the strains (Supplemental Figure 3B), suggesting that this pathway is not a determinant of sensitivity in our model.

### Folate metabolism and transport do not explain strain susceptibility to injury.

The enzyme Dhfr can metabolize low doses of FA to the nontoxic 5,6,7,8-tetrahydrofolic acid. *Dhfr* activity is low in humans but effective in rodents (24). Basal *Dhfr* expression was lowest in the sensitive PWK/PhJ strain but remained high in C57BL/6J, which is also susceptible to injury (Supplemental Figure 4A). Upon FA, *Dhfr* expression was reduced in most strains, but the reduction was lowest in the resistant CAST/EiJ strain and highest in the sensitive C57BL/6J and PWK/PhJ strains (Supplemental Figure 4B). However, this may be a consequence of glomerular injury, rather than a cause. It is unclear how much direct catabolism of FA by *Dhfr* compares with urinary excretion at high doses of FA. To have a more complete picture of folate metabolism across our strains, we measured the expression of proteins involved in mitochondrial folate transport (Supplemental Figure 4C), high-affinity FA transport (25) (Supplemental Figure 4D), folate metabolism downstream of DHFR (26) (Supplemental Figure 4E), and low-affinity folate transporters (25) (Supplemental Figure 4F). All of these genes present a diverse pattern of expression across strains, consistent with the diversity between the represented mouse strains and subspecies. However, these variations have no consistent correlation with the severity of the injury seen in each strain, suggesting that they are not responsible for the observed difference in injury susceptibility or speed of recovery.

### The kidney transcriptome shows an association between early mitochondrial stress and later chronic inflammation.

**Figure 4:**
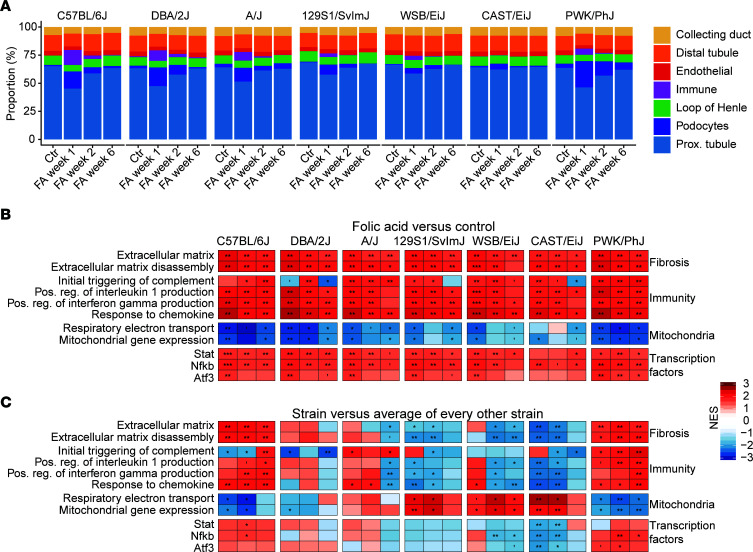
The transcriptional profile reveals immune cell infiltration and activation, along with an early reduction of mitochondrial gene expression in susceptible strains. (**A**) Cell type deconvolution, using the single-cell data from ref. 27. An increase in immune cell proportion, indicative of infiltration, was detected in all strains but the CAST/EiJ, while the relative number of proximal and distal tubule cells is reduced following injury. The increase in proportion of podocytes is surprising and may be an artifact. Fibroblasts were not detected and may have been mislabeled as podocytes. (**B** and **C**) Gene set enrichment analysis of differentially expressed genes. (**B**) Treatment versus control. (**C**) Strain versus the average of all other strains. FDR-corrected *P* values are shown (***P < 0.001, **P < 0.01, *P < 0.05). The comparison between **B** and **C** highlights that, while all strains share the same response (**B**), the relative difference in severity is highly predictive of the clinical outcome (**C**). Specifically, susceptible strains C57BL/6J and PWK/PhJ have higher induction of immune and fibrosis-related genes and higher reduction of mitochondria-related genes.

Given that some of the observed transcriptional differences may be due to changes in the proportions of different cell populations, we performed cell type deconvolution using single-cell data from another study (27) (Figure 4A). We detected an infiltration of immune cells in all strains, except the CAST/EiJ, while the amount of proximal and distal tubule cells was reduced in all strains following injury. We also found an expansion of the proportion of podocytes across all strains (although, once again, greatest in Group III strains), which, to our knowledge, has never been reported before and may be an artifact of the deconvolution procedure, indicating that further inquiry would be required to ascertain this.

We performed a gene set enrichment analysis (GSEA) of the transcriptomic response to kidney injury by comparing either FA with control in each strain (Figure 4B) or by comparing the response in each strain with the response in every other strain (Figure 4C). The comparison with control (Figure 4B) again highlighted how universal the transcriptomic response to AKI was. In all strains and at nearly all time points, even including the resistant CAST/EiJ, we observed an induction of fibrosis and collagen-related gene sets, an induction of immune-related gene sets notably the proinflammatory IFN- and IL-1–mediated responses as well as a reduction in mitochondria-related gene sets. Targets of the critical immune-related NF-κB and Stat families of transcription factors were among the most enriched. A more complete selection of gene sets is also included (Supplemental Figure 5).

GSEA uses the ranking of genes rather than their absolute values, and this ranking is similar in all strains, including the resistant CAST/EiJ, showing that the differences between strains are matters of scale, rather than completely different responses. To better highlight the differences between strains, we performed a differential expression analysis and GSEA on the comparison between each strain and every other strain (Figure 4C). From this, we could infer that the sensitive strains (Group III) differed from recovering strains (Group II) primarily by an early (week 1) downregulation of mitochondria-related gene sets, followed by a late (week 3) activation of immune related gene sets (Figure 4C).

**Figure 5:**
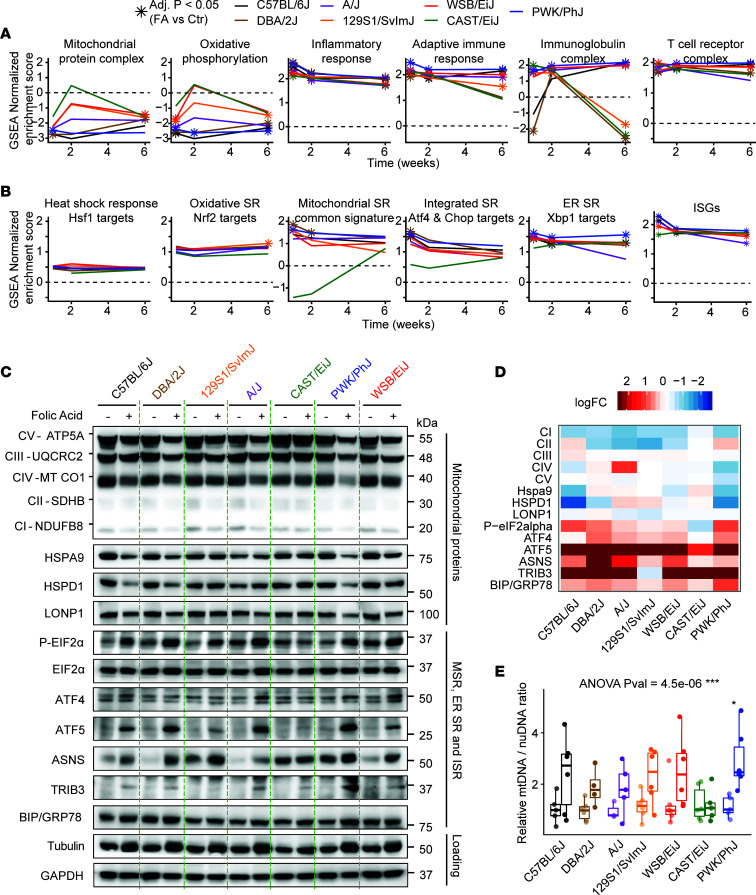
The mitochondrial stress response profile differentiates strains that recover from those that evolve towards chronic disease. (**A**) Selected gene set enrichment analysis (GSEA) across strains. Star-shaped dots indicate adjusted *P* < 0.05 when comparing folic acid to control in a given strain. Compared to other strains, the CAST/EiJ strain loses little mitochondrial activity early and has reduced adaptive immunity later, while the PWK/PhJ strain has strongly repressed mitochondrial activity and later develops persistent T cell– and B cell–mediated adaptive immunity. (**B**) GSEA of major stress pathways using custom gene sets, constructed as described in Supplemental Table 1. The analysis reveals that heat shock and oxidative stress are not significantly induced at any point, while mitochondrial and ER stress are activated throughout, likely feeding into the integrated stress response. SR, stress response. The CAST/EiJ differs very significantly from every other strain (adjusted *P* < 0.0001) when it comes to MSR activation at weeks 1 and 2. (**C**) Western blots from week 1 after folic acid administration and week 2 controls. Mitochondrial respiratory complexes I–V, mitochondrial chaperones, HSPA9, HSPD1, and LONP1; phosphorylation of the central regulator of the integrated stress response, EIF2α, and downstream mediator ATF4; mediators of the MSR, ATF5, ASNS, and TRIB3; and the reporter of ER stress, BIP/GRP78. Tubulin was initially used as a loading control, but it increased upon folic acid administration despite equal amounts of total protein, and GAPDH was used as an alternate loading control. (**D**) Quantification of the western blot from **C**. All proteins were normalized to GAPDH, except P-eIF2α, which was normalized to eIF2α, then fold changes between folic acid and control were computed. (**E**) Measurements of mtDNA/nuclear DNA ratio at week 1 after folic acid administration. The box plot represents median and quartiles. Whiskers reach 1.5 inter-quartile range. ***P < 0.001, **P < 0.01, *P < 0.05, 1-way ANOVA with Tukey post hoc comparisons.

While the overall trend of loss of mitochondrial content and increased inflammation was present in all strains (Figure 5A), their evolution over time strongly differed between Group II and Group III strains. Group II strains lose little mitochondrial gene expression early and then have reduced adaptive immunity later, while sensitive Group III showed a strongly repressed early mitochondrial gene expression and later develop persistent T cell–and B cell–mediated adaptive immunity (most notably the PWK/PhJ). This enhanced expression of adaptive immune response genes, characterized by B and T cell markers, as well as inflammasome activation and *Il1b*, *Il6*, and *Tnf* expression (Figure 3E), has all the features of the low-grade inflammation characteristic of CKD (28, 29).

### Mitochondrial stress responses differ between resistant and sensitive strains.

The transcript levels of the mitokine, *Gdf-15*, were robustly induced in most strains except in CAST/EiJ (Figure 1D and Figure 3E), and this may reflect differences in the induction of the mitochondrial stress response (MSR). To this end, we compared the induction of gene sets involved in the MSR against a wide range of gene sets representative of stress responses in other cellular compartments (Figure 5B). Each stress response was defined by a set of confirmed targets, drawn from overlapped ChIP-Seq and RNA-Seq data sets (Supplemental Table 1). Expression of gene sets indicative of the heat shock and oxidative stress responses were not significantly increased in any of the strains and time points, while transcripts for ER and integrated stress responses (ISR) were induced in most of them, although the induction did not always reach statistical significance (Figure 5B). MSR gene sets increased most at weeks 1 and 2 in all strains but the CAST/EiJ, and this difference between strains was highly significant (adjusted *P* < 0.0001). The gene sets reflecting the ISR were also not significantly enriched in CAST/EiJ mice, likely because it is triggered downstream of both ER and mitochondrial stresses (30), and neither the ER stress or MSR were induced in this strain. IFN-stimulated genes (ISGs) were also activated across all strains (Figure 5B). This immune response is also known to be activated through the cGAS-STING pathway by mitochondrial DNA (mtDNA) released by damaged or stressed mitochondria (31, 32) or released after an injury (33).

To assess whether the MSRs that we observed were also present at the protein level, we performed Western blot analysis of key mitochondrial oxidative stress components — mediators of the MSR, ISR, and ER stress — and downstream targets at the early time point, 1 week after kidney injury (Figure 5C) and also performed quantification (Figure 5D). All strains except the CAST/EiJ strain had reduced expression of mitochondrial electron chain (complexes I–V) and chaperone proteins (HSPA9, HSPD,1 and LONP1), and this loss was most pronounced in the PWK/PhJ strain (Figure 5, C and D). The central pathway of the ISR proceeds from EIF2α phosphorylation, leading to translation blockade and preferential translation of select proteins, most notably the translation factor ATF4 (34). Both EIF2α phosphorylation and ATF4 were induced in most strains, testifying to the activation of the ISR (Figure 5, C and D). The mediators of the MSR, ATF5, ASNS, and TRIB3 were strongly increased (35), while BIP/GRP78, an indicator of ER stress, was only modestly increased (Figure 5, C and D). Interestingly, the mtDNA/nuclear DNA ratio significantly increased across strains (1-way ANOVA, *P* < 0.001), although it was only individually significant in the PWK/PhJ strain (Figure 5E) and there was no increase in the CAST/EiJ. The basal levels of mitochondrial proteins (Figure 5C) and mtDNA/nuclear DNA ratio (Figure 5D) showed little variation between strains at baseline, indicating that the mitochondrial quantity before injury was similar at baseline. This increase in mtDNA in damaged or stressed mitochondria is consistent with several studies that found accumulation of mtDNA in damaged or dysfunctional mitochondria (36–39).

Across all these measurements, the PWK/PhJ strain was notable for the more pronounced loss of mitochondrial proteins and robust activation of the MSR, while the CAST/EiJ was almost nonresponsive. The CAST/EiJ strain exhibited a slight increase in BIP/GRP78, reflecting the induction of ER stress genes (Figure 5, C and D). The lack of induction of mitochondrial stress, but not other cellular stress pathways, is a unique feature of the CAST/EiJ and may play a role in its resistance to injury.

### Early loss of NAD^+^ synthesis and salvage, and increased consumption of NAD^+^, are early events that predict disease severity.

**Figure 6:**
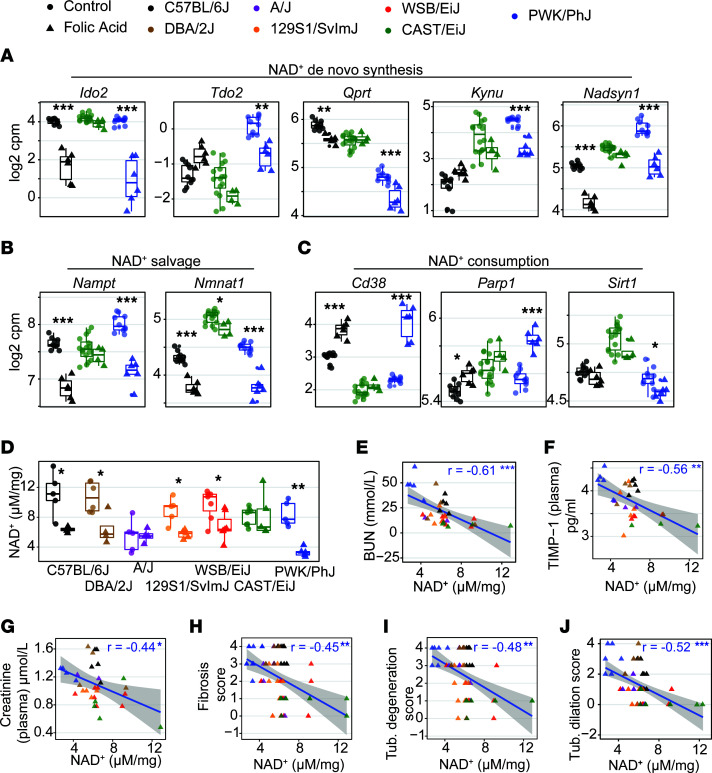
Transcripts of NAD^+^ biosynthesis are downregulated, while those of NAD^+^ consumption increase in susceptible strains, and this is reflected by changes in NAD^+^ levels and disease severity. (**A**–**C**) Box plot of RNA-Seq–based gene expression of NAD^+^ de novo synthesis (**A**), NAD^+^ salvage (**B**), and NAD^+^ consumption (**C**) genes in the 3 most stereotypical strains after 1 week of folic acid administration. Other strains are summarized on Supplemental Figure 6. While the CAST/EiJ strain undergoes little variation upon FA treatment, in the PWK/PhJ and to a lesser extent in the C57BL/6J strain, there was a marked reduction in transcript expression of NAD^+^ biosynthesis accompanied by an increase in transcripts of the NAD^+^ consumers, *Cd38* and *Parp1*, while *Sirt1* underwent little variation. The box plot lower and upper hinges correspond to the first and third quartiles, and the center line is the median. The whiskers extend from the hinge to the largest value no further than 1.5***×*** the interquartile range. FDR-adjusted *P* values are shown (***P < 0.001, **P < 0.01, *P < 0.05), using moderated 2-tailed *t* test. (**D**) Kidney NAD^+^ levels measured by HPLC-MS. This confirms that the reduction of NAD^+^ content is strongest in the PWK/PhJ strain and absent in the CAST/EiJ and A/J strains. FDR-adjusted *P* values are shown (***P < 0.001, **P < 0.01, *P < 0.05), using moderated 2-tailed *t* test. (**E**–**J**) Correlation between NAD^+^ levels and blood parameters, blood urea nitrogen (**E**), TIMP-1 (**F**), creatinine (**G**), and the kidney histology scores for fibrosis (**H**), tubule degeneration (**I**), and tubule dilation (**F**). The blue line is a linear fit of the data. Statistics significance is shown with Pearson *r* and FDR-corrected *P* values (***P < 0.001, **P < 0.01, *P < 0.05), using Pearson correlation test, corrected for multiple testing over all possible phenotype comparisons. These correlations and those pictured Supplemental Figure 7 indicate that NAD^+^ levels may be highly predictive of AKI susceptibility in mice.

The coenzyme nicotinamide adenine dinucleotide (NAD^+^) is critical for mitochondrial function and interventions on NAD^+^ have been shown by us and others to have wide-ranging beneficial effects on mitochondrial-related diseases (reviewed in ref. 40). In addition, several recent studies have implicated NAD^+^ metabolism in both chronic and acute forms of kidney disease — AKI (41–44), IRI (45) and diabetic kidney disease (46). Furthermore, the central mediator of the ISR DDIT3/CHOP — which is downstream of both MSR and ER stress responses — was implicated as a potential regulator of NAD^+^ synthesis in AKI through the critical synthesis enzyme QPRT (43). Given the strong differences observed in the MSR and ISR pathways in our study, we assessed the transcript levels of the critical enzymes involved in NAD^+^ de novo synthesis (Figure 6A and Supplemental Figure 6A), salvage (Figure 6B and Supplemental Figure 6A), and consumption (Figure 6C and Supplemental Figure 6A). While the CAST/EiJ strain undergoes almost no variation in expression of NAD^+^-related transcripts, the PWK/PhJ and, to a lesser extent, the C57BL/6J strain underwent a significant loss of NAD^+^ biosynthesis genes accompanied by a large increase in the NAD-consuming genes *Cd38* and *Parp1*. Other strains presented an intermediate phenotype (Supplemental Figure 6A), and NAD^+^ synthesis genes were mostly restored at week 2, except in the PWK/PhJ and C57BL/6J strains, while only the PWK/PhJ strain retained reduced *Qprt* and increase *Cd38* at week 6 (Supplemental Figure 6A).

We then assessed NAD^+^ levels by high-performance liquid chromatography–mass spectrometry (HPLC-MS) and found that it was reduced at week 1, with a 3-fold reduction in the PWK/PhJ strain and lesser reductions in other strains except the CAST/EiJ and A/J strains (Figure 6D). NAD^+^ levels remained lower at weeks 2 and 6 but were only significant in a few strains (Supplemental Figure 6B). Baseline NAD^+^ levels (Figure 6D) and NAD^+^-related genes (Figure 6, A–C, and Supplemental Figure 6A) varied strongly across strains, but these baseline differences before injury had no obvious relation to resistance to injury. However, NAD^+^ levels upon injury across strains negatively correlated with biochemical (Figure 6, E–G, and Supplemental Figure 7A) and histological (Figure 6, H–J, and Supplemental Figure 7A) markers of disease severity, and many of these correlations were maintained even at weeks 2 and 6 (Supplemental Figure 7, B and C), indicating that early loss of NAD^+^ may be a biomarker of disease severity and duration and that the PWK/PhJ strain may be a suitable model for the role of NAD^+^ in human kidney diseases.

### The mouse transcriptomic response to FA matches human nephropathies.

**Figure 7:**
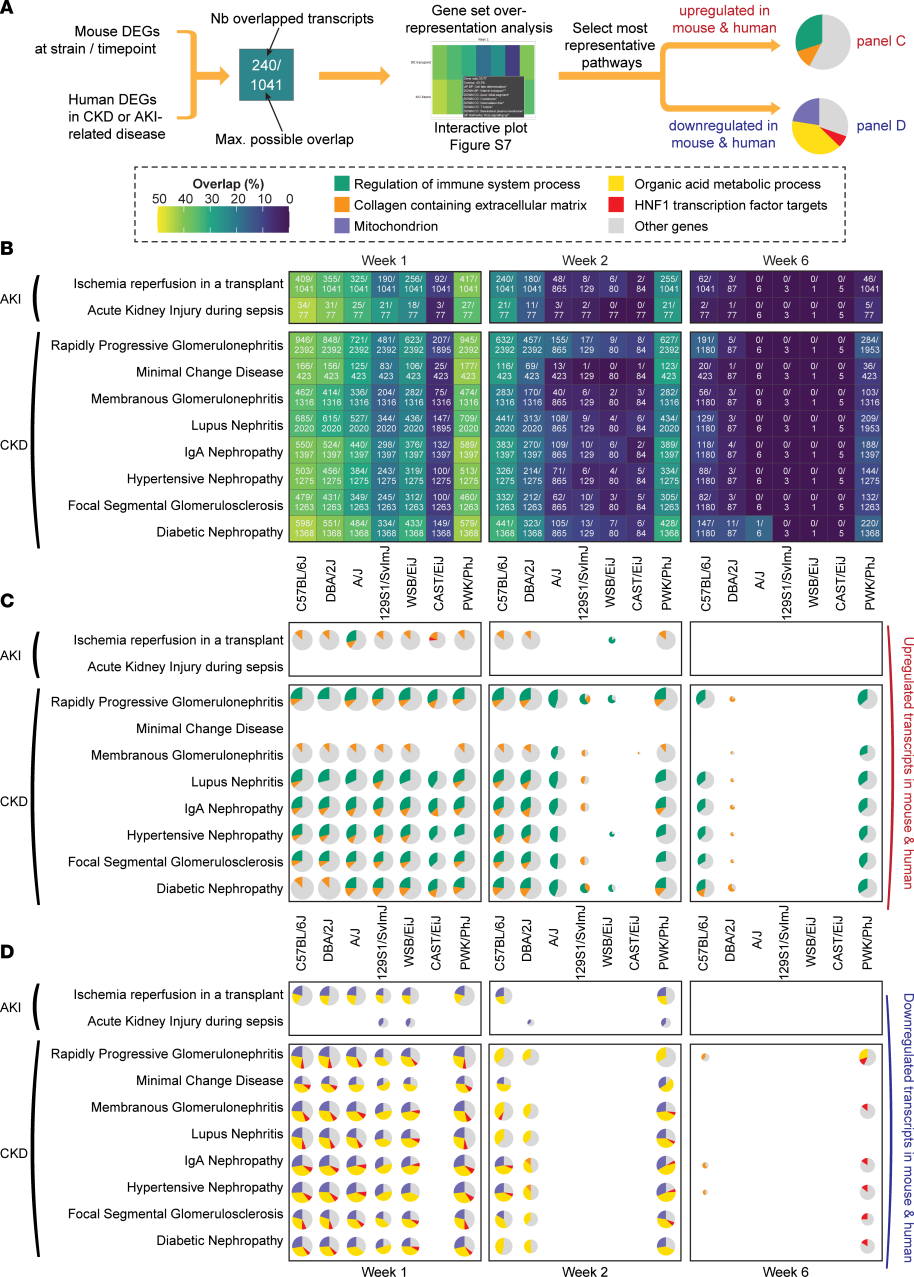
The transcript profile of the CC founder strains matches that of both CKD and AKI at week 1, while the PWK/PhJ and C57BL/6J strains match CKD at 6 weeks. (**A**) Schematic of the approach. Gene expression signatures of human kidney diseases were obtained from public repositories (see Supplemental Table 2) and then compared with the mouse differentially expressed genes upon folic acid treatment. Both human and mouse data sets used a differentially expressed genes (DEGs) cutoff of |log_2_(fold change)| > 0.5 and FDR-corrected *P* < 0.05. Transcripts were considered overlapping if they varied in the same direction in mice and humans. Overlap percentage is “number overlapped transcripts”/“maximum possible overlap”, where the maximum possible overlap is the number of human and mouse DEGs — whichever is smallest. For each overlap, we performed a GSEA (see Supplemental Figure 8). (**B**) Overlap plot at week 1, exact numbers are indicated within each tile. Human kidney diseases overlap strongly with the response to folic acid in mice. This concordance was lost in most strains at week 6, except in the PWK/PhJ and C57BL/6J, which retained a large overlap (~20% genes) with human chronic kidney diseases. (**C** and **D**) Pie charts of selected significant gene sets among upregulated (**C**) or downregulated (**D**) transcripts. The size of each pie chart is log-proportional to the number of overlapped genes. The greatest overlaps involved an upregulation of immune-related transcripts and extracellular matrix components and a downregulation of mitochondrial and metabolic transcripts. This indicates a strong similarity between fibrogenesis, immune, and metabolic pathways implicated in mice and humans. At week 6, the inflammatory signature in the PWK/PhJ and C57BL/6J strains was similar to chronic human diseases but lost their similarities with acute kidney disease.

To address the relevance of these results to human pathologies, we used a set of gene signatures from analyses of a range of different human nephropathies, representing the transcriptomic response in both acute and chronic forms of kidney disease (Supplemental Table 2). For each human disease, we first examined the degree of overlap between differentially expressed genes in humans and those in our study in every strain and time point; we then performed pathway overrepresentation analyses to understand which pathways were shared between mice and humans (Figure 7A). At week 1, there was a strong overlap between FA-treated mice and human diseases representative of either CKD or AKI (Figure 7B), which was surprising considering that the AKI and CKD data sets we used had little overlap with each other (Supplemental Figure 9A); this overlap suggests that the mice at week 1 after FA may already have a phenotype somewhere between CKD and AKI. The overlap was strongest in PWK/PhJ and C57BL/6J mice, with — most often — between 40% and 50% of shared DEGs between mice and humans (Figure 7B). This validates our model of low-dose FA injury and subsequent recovery as highly representative of human disease. At week 6, the similarity between mouse DEGs and human AKI DEGs was mostly lost, while the PWK/PhJ and C57BL/6J strains retained a strong similarity to human CKD (Figure 7B), indicating that they may have transitioned from AKI to CKD.

### Critical pathways are shared between human kidney diseases and the FA response in C57BL/6J and PWK/PhJ strains.

To assess which biological pathways were most shared between our mouse model and humans, we performed an overrepresentation analysis for each of the mouse condition–human disease pairs (Figure 7A and Supplemental Figure 8). Among the 5 most represented pathways, strains “regulation of immune system process” (green), representing immune activation, and “collagen-containing extracellular matrix” (orange), representing fibrogenesis, were consistently upregulated in mice and humans (Figure 7C), highlighting that the same genes are implicated in these pathway activations across species. Immune activation, but not fibrogenesis transcripts, was maintained at week 6 in the C57BL/6J and PWK/PhJ strains (Figure 7C).

Among shared downregulated genes, “mitochondrion”, and “organic acid metabolic process” represented up to 3 quarters of shared downregulated transcripts between mice and human CKD at weeks 1 and 2, highlighting the absolutely central role of mitochondria and metabolism in kidney disease (Figure 7D). This loss of metabolic genes was stronger in CKD than AKI-related diseases, suggesting that our model may be especially suited to modelling metabolic alterations in CKD. An unexpected finding was a strong enrichment for downregulated targets of the transcription factor HNF1 (red; (Figure 7D), which is present in most strains at week 1 but is strongly increased in the PWK/PhJ strain at week 6. The HNF1 family of transcription factors consists of 2 members, HNF1α and HNF1β, which form heterodimers to regulate lipid and metabolic genes (47). Mutations in Hnf1β cause renal cysts and renal function decline in both humans and mice (48, 49). Similarly, *Hnf1a* variants were picked up in a recent genome-wide association studies (GWAS) for kidney function with > 1.2 million patients (50). Our data, however, point to downregulation of HNF1 targets as a shared feature in many kidney diseases, and the PWK/PhJ strain may be especially suited to study this. The expression of *Hnf1a* and *Hfn1b* genes was reduced in both C57BL/6J and PWK/PhJ strains at week 1, and a trend toward downregulation was still present at week 6 in the PWK/PhJ (Supplemental Figure 9B). This comparison with humans highlights the validity of our mouse model of acute injury and transition to chronic disease, and it points to the PWK/PhJ strain as a promising model to study the role of both NAD^+^ and HNF1 in human kidney disease and the AKI-to-CKD transition.

### A web resource on kidney disease.

The phenotypic traits and transcriptome data collected in this study can be explored with an online, interactive interface (www.systems-genetics.org/CC_founders_AKI). This resource enables researchers to examine the individual variation of FA-induced injury in all mouse strains and to choose an appropriate mouse model.

## Discussion

In the clinic, the onset of AKI is often unpredictable; no one can anticipate a hemorrhagic shock, severe burn, or adverse reaction to antibiotics, but with the increased penetrance of personalized medicine, knowing one’s genetic predisposition to AKI and whether one will fully recover from this injury and avoid development of progressive fibrosis or CKD may become a reality in the near future. Our study was designed to emulate differences in genetic susceptibility on the clinical progression from AKI to interstitial fibrosis and CKD through a diverse panel of mouse strains. Across the 7 mouse strains, whether we consider the plasma and urine markers, organ weight changes (Figure 1), or histological markers (Figure 2), we observed a continuum of severity from “highly susceptible” (C57BL/6J, PWK/PhJ, DBA2/J) over “recovers well” (A/J, 129SvlmJ, WSB/EiJ) to “fully resistant” (CAST/EiJ).

Mitochondrial defects are one of the earliest events observed in human AKI (51), and defects in mitochondria have been reported in FA nephropathy mouse models and have been proposed as either potential biomarkers (42, 52) or therapeutic targets (42, 53). A distinguishing feature of the resistant CAST/EiJ strain compared with more susceptible strains was the absence of the induction of the MSR. This difference in MSR in the CAST/EiJ was confirmed at the protein level and was supported by a lack of GDF-15 induction, a mitokine known to signal mitochondrial stress and influence CKD progression (20, 54) (Figure 1D and Figure 3E). FA can directly be taken up by mitochondria and cause mitochondrial damage, and the kidney toxicity of FA was shown to be prevented by N-acetyl-cysteine before administration (55). This direct effect of FA on mitochondria can be a potential confounder in our model. However, the mitochondrial defects we observed strongly overlap decreased mitochondrial genes in human CKD, which suggests that this model remains relevant to human disease. In our comparison with human disease, the CAST/EiJ strain shared an early induction of immunity and fibrogenesis (Figure 7C) but lacked the concomitant reduction in mitochondrial and metabolism transcripts (Figure 7D).

The critical metabolic coenzyme NAD^+^ is increasingly recognized as a central modulator of both mitochondrial-related diseases (40) and acute and CKD (41–46). GWAS have also revealed different kidney susceptibility associated with NAD^+^-related genes. The rate-limiting NAD^+^ salvage enzyme NAMPT was associated with serum creatinine and eGFR in one UK biobank–based GWAS (56), and SIRT-1 was associated with BUN in a cross-population study (57) (accessed through the NHGRI-EBI GWAS catalog; ref. 58), highlighting the importance of this pathway in humans. Although we cannot conclude on whether loss of NAD^+^ was the cause or the consequence of kidney damage, NAD^+^ levels at an early time point were indicative of both disease severity and duration. Our results further suggest that MSR genes, mitokines such as GDF-15, and metabolic-related transcription factors such as HNF1 may be of particular interest as both biomarkers and targets in early AKI.

The association between early mitochondrial stress and prolonged inflammation evident at later stages after the initial insult may be linked to the release of mitochondrial DAMPs (33) and the activation of cGAS-STING and IFN signaling feeding into the inflammatory NF-κB pathway — features that were also evident in our susceptible strains. Genetic studies of the susceptibility to AKI point to a complex multigenic environment, but inflammatory genes — chiefly *TNFa, Il6, STAT1* and *NFKB-1* — are among the most important genetic determinants (5). In mouse strains, *Tnf-a* and *Il6* were highly correlated with the severity of the injury (Figure 3E), as were enrichments of NF-κB and Stat targets (Figure 4C), highlighting similar trends across species. Of note, such immune regulation genes were shared between the PWK/PhJ strain and most forms of human CKD at week 6 (Figure 7B). The PWK/PhJ strain was particularly notable for its prolonged and intense upregulation of the Nlrp3 inflammasome, which remains strongly upregulated at week 6 (Figure 3E). In mice, Nlrp3 inflammasome activation in macrophages is thought to be sufficient to trigger chronic inflammation (59), and its strong upregulation specifically in the PWK/PhJ strain may be a further indicator of a transition toward chronic disease. Our results point particularly to early mitochondrial stress and reduced NAD^+^ content, as well as the mitokine GDF-15, as potential translatable biomarkers of severe AKI, which may be of interest in humans.

Our collection of phenotypic and molecular traits can be explored in an online resource (www.systems-genetics.org/CC_founders_AKI). This resource can help researchers select the ideal animal model to study particular aspects of AKI, but it also showcases the importance of examining the diversity of outcomes present across mice before attempting translation to humans, as findings originating from a single mouse strain often translate poorly to humans (7). From resistance to kidney injury in the CAST/EiJ strain (in humans, only 12% of surgery patients develop AKI; ref. 60), to reversible AKI (Group II strains) and transition to chronic disease and inflammation (Group III strains, and chiefly the PWK/PhJ strain), the continuum of responses to AKI and development of kidney fibrosis following AKI in this study is a good basis for understanding which mechanisms lead to disease progression and guide therapeutic efforts in the right direction. In addition, by looking at the extreme phenotypes of the resistant CAST/EiJ and sensitive PWK/PhJ strain, our results make the case that mitochondrial and metabolic biomarkers such as NAD^+^ are critical determinants of recovery after AKI or progression to CKD.

## Methods

### Choice of mouse models.

This study used 7 domesticated (C57BL/6J, DBA/2J, A/J, 129S1/SvlmJ, and WSB/EiJ) or wild-derived (CAST/EiJ and PWK/PhJ) inbred mouse strains drawn from founders of the well-characterized BXD and CC panels, which are well known for their diversity in genetics, as well as in molecular and cardiometabolic phenotypes (61). The CC founder strains NOD/ShiLtJ and NZO/HlLtJ were excluded because they naturally develop diabetes and other symptoms in the absence of injury (61) (NOD, diabetes and immune defects; NZO, severe obesity and diabetes), and this causes hyperfiltration and could interfere with the conclusions of the study.

### Mouse handling.

Mouse strains were imported from Charles River and bred at the EPFL animal facility for more than 2 generations before incorporation into the study. The mice were fed a chow diet (Harlan 2018; 6% kCal of fat, 20% kCal of protein, and 74% kCal of carbohydrates). Mice were housed at 2–4 animals per cage under 12-hour light/dark cycle, with ad libitum access to food and water at all times. BW was measured weekly from 8 weeks of age until killing. We examined 7 mouse inbred strains. At 9 weeks of age, 3 groups of mice were treated with 125 mg/kg of FA in 3M sodium bicarbonate, and 2 groups were treated with bicarbonate alone (vehicle controls). Strains were entered into each group randomly and were then observed daily to monitor their health. The mice were scored weekly according to BW/food intake, coat condition, movement, and signs of pain. Mice scoring above 1 were monitored 3 times a week, and mice scoring above 2 were monitored daily. Mice with a score of 3 were sacrificed.

### Sacrifices and sample collection.

Mice were sacrificed at week 1, 2, and 6 (FA treated) or weeks 2 and 6 (controls). The sacrifices took place from 8:30 a.m. until 12 p.m., with isoflurane anesthesia followed by a complete blood draw (~1 mL) from the vena cava and by perfusion with phosphate-buffered saline. Half of the blood was placed into lithium-heparin–coated (LiHep-coated) tubes and the other half in EDTA-coated tubes. Then, both were shaken and stored on ice, followed immediately by collection of the kidneys, liver, heart, spleen, gastrocnemius, and epididymal white adipose tissue. The LiHep blood taken for plasma analysis was also centrifuged at 3,600*g* at 4°C for 10 minutes at 4°C before being flash-frozen in liquid nitrogen. The left kidney and other organs were flash-frozen in liquid nitrogen and stored at –80°C until processing. The right kidney was split according to the schematic Supplemental Figure 10, and parts were stored in formalin or OCT for histological analysis.

### Urine and plasma biochemistry.

Plasma from LiHep-coated tubes was stored at –80°C prior to analysis. Plasma parameters were measured 2 times on diluted samples (1:1 ratio of plasma to diluent) using Dimension Xpand Plus (Siemens Healthcare Diagnostics AG). The biochemical tests were performed according to the manufacturer kit for each parameters: enzymatic creatinine (Siemens Healthcare, DF270B), glucose (Siemens Healthcare, DF40), transaminase ASAT (Siemens Healthcare, DF41A), transaminase ALT (Siemens Healthcare, DF143), and urea nitrogen (Siemens Healthcare, DF21). Plasma levels of TIMP-1, FGF-21, and GDF-15 were measured using the Mouse Premixed Multi-Analyte Kit (LXSAMSM, R&D Systems) in a Luminex 200 system following manufacturer’s instructions.

### Histopathology.

With more than 48 hours of formation fixation, the selected middle cross sections of the right kidneys from all mice were then rinsed in 70% ethanol, trimmed, and processed with a conventional paraffin-embedding technique. Paraffin-embedded specimens were then blocked and sliced using rotary microtome at 5 μm thickness; then, sliced sections were stained either with H&E or Picrosirius red (PSR) using internal protocols. All slides were then evaluated by an experienced pathologist in a double-blinded manner. A 20% gradient scoring method based on percentage of affected region of lesions was applied as the semiquantitative analysis assay (Grade 1, < 20%; Grade 2, 21%–40%; Grade 3, 41%–60%; Grade 4, 61%–80%; Grade 5 > 81%).

### Quantitative kidney tissue section image analysis for collagen content.

Automated tissue section–based quantification of PSR histochemical staining (surrogate for collagen) was performed using image analysis algorithms in Visiopharm (version 2020.08.0.8126, Visiopharm). Cortex, medulla, and renal papilla were manually annotated based on morphological criteria from whole transversal kidney sections. Pelvis and adjacent connective tissues were excluded from the analysis.

### RNA extraction.

For mRNA, liver tissues were crushed in liquid nitrogen, and 10 mg of tissues were suspended in TRIzol (Invitrogen) and homogenized with stainless steel beads using a TissueLyser II (Qiagen) at 30 Hz for 2 minutes. RNA was extracted and purified using Direct-zol-96 RNA kits (Zymo Research). mRNA concentration was measured for all samples. All samples passed a quality check of purity (NanoDrop) and fragmentation (FragmentAnalyzer).

### RNA-Seq and mapping.

RNA libraries were prepared for sequencing using SMARTER mRNA-Seq Library Prep Kit standard protocols. RNA-Seq was performed on a BGISEQ-500. FastQC (default parameters) was used to verify the quality of the mapping. No low-quality reads were present, and no trimming was needed. The STAR aligner (62) was used for mapping the RNA-Seq data to the C57BL/6J reference genome and determining gene counts. We did not use distinct genomes for each strain due to various genome-quality differences between mouse strains that could create bigger artefacts than mapping all strains on the same reference genome, in terms of mapping efficiency and gene count estimation.

### Comparison with human data sets.

Human CKD signatures were downloaded from ref. 63, and AKI data sets were downloaded from the GEO database as indicated on Supplemental Figure 2 (64, 65). For CKD, we used the DEGs provided (63), whereas for AKI data sets, we downloaded the data from raw data from GEO and performed differential gene expression using the limma R package and the voom method (66). For all data sets, we selected the DEGs with |fold change| > 0.5 and FDR < 0.05. Genes were only considered overlapping if they varied in the same direction in both mice and humans. The overlap percentage was calculated as “number overlapped genes”/”maximum possible overlap”, where the maximum possible overlap is the number of human and mouse DEGs — whichever is smallest. For each set of common genes between mice and humans, we performed a gene set overrepresentation analysis using the clusterprofiler R package (67) and represented it as an interactive visualization (Supplemental Figure 8). Five of the most commonly overrepresented gene sets were selected and were represented as pie charts, where the sections of the pie represent the proportion of genes in a given gene set.

### Western blots.

Frozen kidney samples, pooled from 4 randomly picked mouse kidneys for each condition, were lysed by mechanical homogenization with RIPA buffer containing inhibitors for protease (catalog 78430, Thermo Fisher Scientific) and for phosphatase (catalog 78428, Thermo Fisher Scientific). The concentration of extracted protein was then determined and normalized using the DC Protein Assay Reagents (catalog 5000116, Bio-Rad). Subsequently, the lysates were analyzed by SDS–PAGE and Western blots using the following antibodies: Total OXPHOS Cocktail (catalog ab110413, Abcam, 1:1,000), P–Eif-2α (catalog 3597, CST, 1:500), Eif-2α (catalog 9722, CST, 1:1,000), Atf5 (catalog ab60126, Abcam, 1:1,000), Atf4 (catalog 11815, CST, 1:1,000), Asns (catalog sc-365809, Santa Cruz Biotechnology Inc., 1:1,000), Hspa9 (catalog ABIN361739, Antibodies Online, 1:1,000), Hspd1 (catalog sc-59567, Santa Cruz Biotechnology Inc., 1:1,000), Lonp1 (catalog HPA002192, MilliporeSigma, 1:1,000), Trib3 (catalog 66702-1, Proteintech, 1:1,000), BIP/Grp78 (catalog ab21685, Abcam, 1:1,000), Gapdh (catalog sc-365062, Santa Cruz Biotechnology Inc., 1:1,000), Tubulin (catalog T5168, MilliporeSigma, 1:2,000), and HRP-labeled anti-rabbit (catalog 7074, CST, 1:5,000) and anti-mouse (catalog 7076, CST, 1:5,000) secondary antibodies. See complete unedited blots in the supplemental material.

### MtDNA/nuclear DNA ratio.

The mtDNA/nuclear DNA ratio was measured as described in ref. 68. Briefly, DNA was extracted from crushed kidney by isopropanol precipitation in 0.3M sodium acetate, followed by washing in 70% ethanol. Quantitative PCR (qPCR) was performed on a Lightcycler 480 II (Roche) with the following primers: 16rRNA Fwd (5′–3′): CCGCAAGGGAAAGATGAAAGAC, Rev: TCGTTTGGTTTCGGGGTTTC; ND1 Fwd: CTAGCAGAAACAAACCGGGC, Rev: CCGGCTGCGTATTCTACGTT; HK2 Fwd: GCCAGCCTCTCCTGATTTTAGTGT, Rev: GGGAACACAAAAGACCTCTTCTGG; and UCP2 Fwd: CTACAGATGTGGTAAAGGTCCGC, Rev: GCAATGGTCTTGTAGGCTTCG. The relative mtDNA/nuclear DNA ratio was computed by the ΔΔCt method.

### Kidney NAD^+^ measurements.

NAD^+^ was extracted using the acidic extraction method and was analyzed by HPLC-MS as described (69). Briefly, approximately 10 mg of frozen crushed kidney was used for NAD^+^ extraction in 10% perchloric acid and neutralized in 3M K_2_CO_3_ on ice. After final centrifugation (50,000*g* at 4°C for 5 minutes), the supernatant was filtered and the internal standard (NAD^+^-C13) was added and loaded onto a column (Kinetex 2.6 µm EVO C18 100 Å, LC Column 150 ***×*** 2.1 mm). HPLC was run for 1 minute at a flow rate of 300 mL/min with 100% buffer A (methanol/H_2_O, 80/20% v/v). Then, a linear gradient to 100% buffer B (H_2_O + 5 mM ammonium acetate) was performed (at 1–6 minutes). Buffer B (100%) was maintained for 3 minutes (at 6–9 minutes), and then a linear gradient back to 100% buffer A (at 9–13 minutes) started. Buffer A was then maintained at 100% until the end (at 13–18 minutes). NAD^+^ eluted as a sharp peak at 3.3 minutes and was quantified on the basis of the peak area ratio between NAD^+^ and the internal standard and normalized to tissue weight and protein content.

### Interactive data visualization and availability.

All metabolic traits, mitochondrial activity, and transcriptome data collected in this study can be explored with an online, interactive interface at www.systems-genetics.org/CC_founders_AKI

RNA-Seq data from this study have been submitted to GEO database (GSE222570; https://0-www-ncbi-nlm-nih-gov.brum.beds.ac.uk/geo/query/acc.cgi?acc=GSE222570), and phenotype data from this study is available through the online app.

### Statistics.

All the bioinformatics and statistical analyses were performed in R 3.5.2 and Rstudio Pro. All performed statistical tests were 2-sided. When needed, *P* values were corrected for multiple testing with the Benjamin-Hochberg FDR. Histology scores were compared using Wilcoxon test due to being nonlinear values. mtDNA/nuclear DNA ratios were compared with 1-way ANOVA. Differential gene expression was done using the limma R package and the voom method (66). Cell type deconvolution was performed using MuSiC (70). GSEA was done using the GSEA method of the clusterprofiler R package (67). Genes were ranked according the signed –log_10_ (Benjamin-Hochberg–adjusted *P* value) obtained by looking at the treatment effect in female or male mice in each strain separately . We selected the gene sets from the biological process of the gene ontology that had the highest significance levels in the overall response to the diet in each sex. Plots used the ggplot2 (71) and plotly (72) R packages.

### Study approval.

Mouse experiments were approved by the Swiss cantonal veterinary authorities of Vaud under license 30759. All human data used are anonymized and publicly available.

## Author contributions

JDM, MBS, SM, MB, DR, RAGV, and JA conceived and designed the project. DH performed animal experiments together with technicians and animal facility personnel. TYL and JDM performed laboratory experiments. EB prepared histology samples, JYM and VC performed PSR quantitation, and TC completed histopathology analysis. JDM, MBS, AMB, and NA analyzed the data. MBS and GVA created the web interface. JDM and JA wrote the manuscript with input from all the authors.

## Supplementary Material

Supplemental data

Supplemental Figure 8

## Version 1 02/08/2023

Electronic publication
