# Supplementary material for: Mitochondrial and NAD+ metabolism predict recovery from acute kidney injury in a diverse mouse population
Source: JCI Insight. 2023 Feb 8;8(3):e164626. doi: 10.1172/jci.insight.164626 (PMC9977436; doi:10.1172/jci.insight.164626)
Supplement: Supplemental data [file jciinsight-8-164626-s215.pdf]

Supplementary Materials

Mitochondrial and NAD+ metabolism predict recovery from acute kidney injury in a diverse mouse population

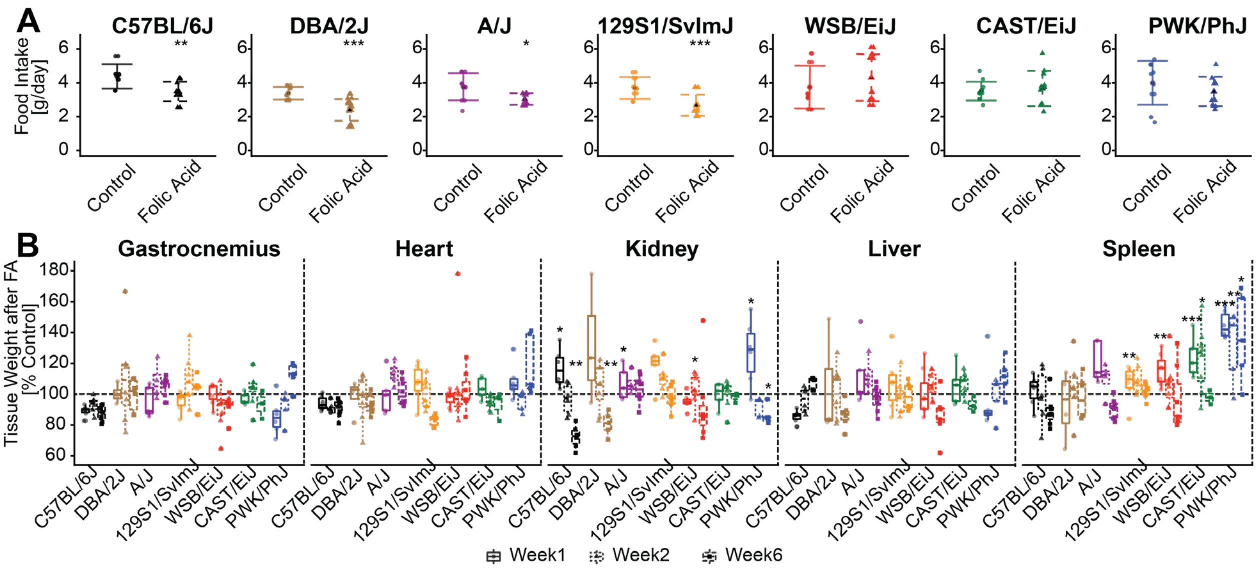

Figure S1: Phenotype evolution of FA injury

A: Food intake per cage and strain, one week after folic acid or vehicle injection.

B: Tissue weights, expressed as percentage of controls across strains and conditions. Student t-test pvalues ***<0.001, **<0.01,*<0.05

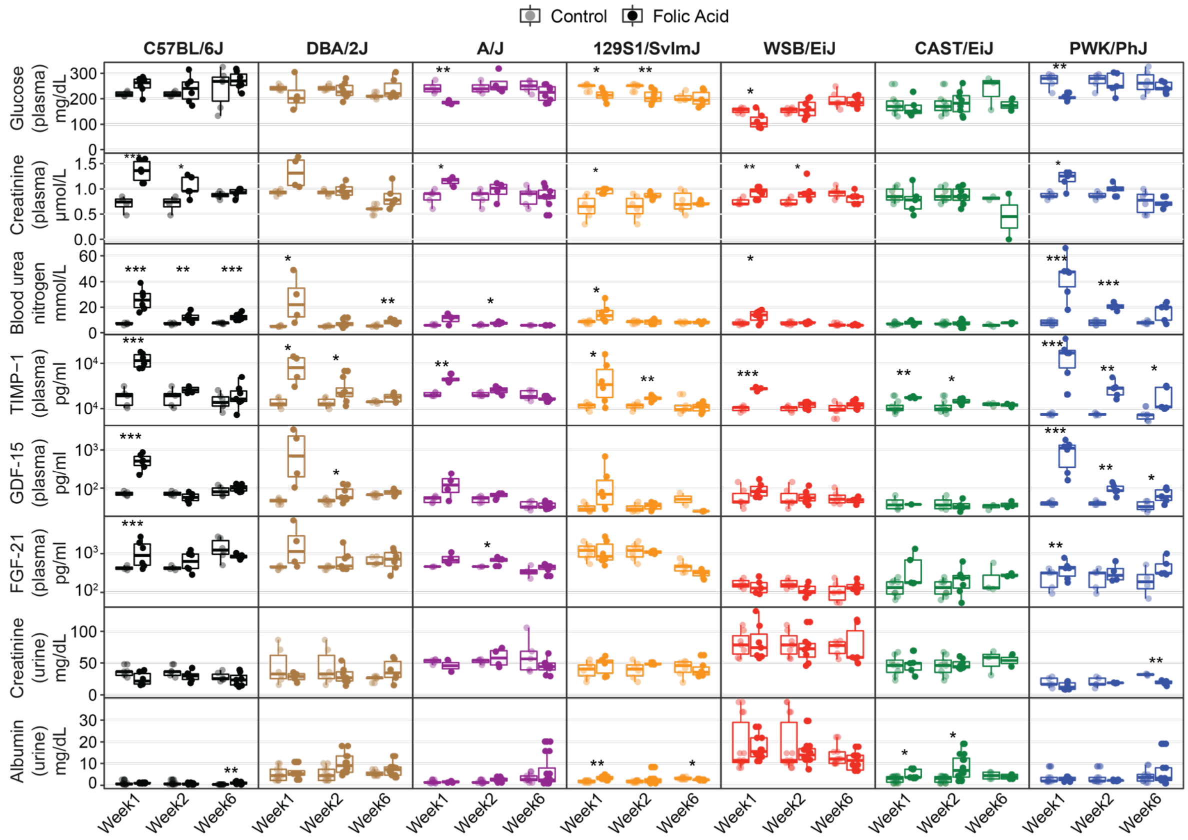

Figure S2: Overview of the biochemistry results in plasma and urine: Boxplots of the urine and plasma biochemistry results. In addition to the effects of folic acid, strains exhibit strong baseline differences in some parameters, such as the WSB/EiJ strain having low blood glucose and high urine albumin at baseline. The PWK/PhJ and C57BL/6J strain emerge as clear outliers in their response to folic acid in most parameters. Note that plasma circulating factors TIMP-1, GDF-15 and FGF-21 are represented on a log10 scale. Student t-test p values, adjusted for multiple testing by BH-method ***<0.001, **<0.01,*<0.05. This data can be explored in further detail through our online interface at www.systems-genetics.org/CC_founders_AKI.

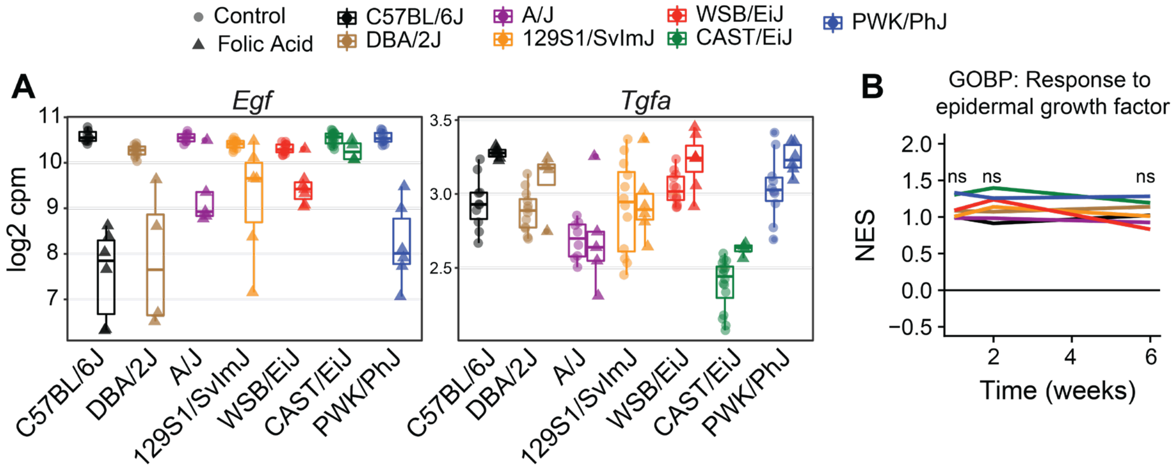

Figure S3: Gene expression and gene set enrichment of *Egf* and *Tgfa*.

A: Log counts per million (cpm) of the gene expression of *Egf* and *Tgfa*. Egf basal expression is near-constant between the strains and decreases strongly upon injury in all strains but the CAST/EiJ, consistent with reports in multiple kidney diseases. Conversely, expression of *Tgfa* was more variable between strains, both at baseline and upon injury.

B: Gene set enrichment of EGF/TGFα targets over time based on the GOBP gene set "Response to Epidermal growth factor" Despite the changes in *Egf* and *Tgfa* expression, no significant enrichment or difference between strains are observed. *ns*: not significant, FDR-corrected Pvalue of gene set enrichment test over GOBP gene sets.

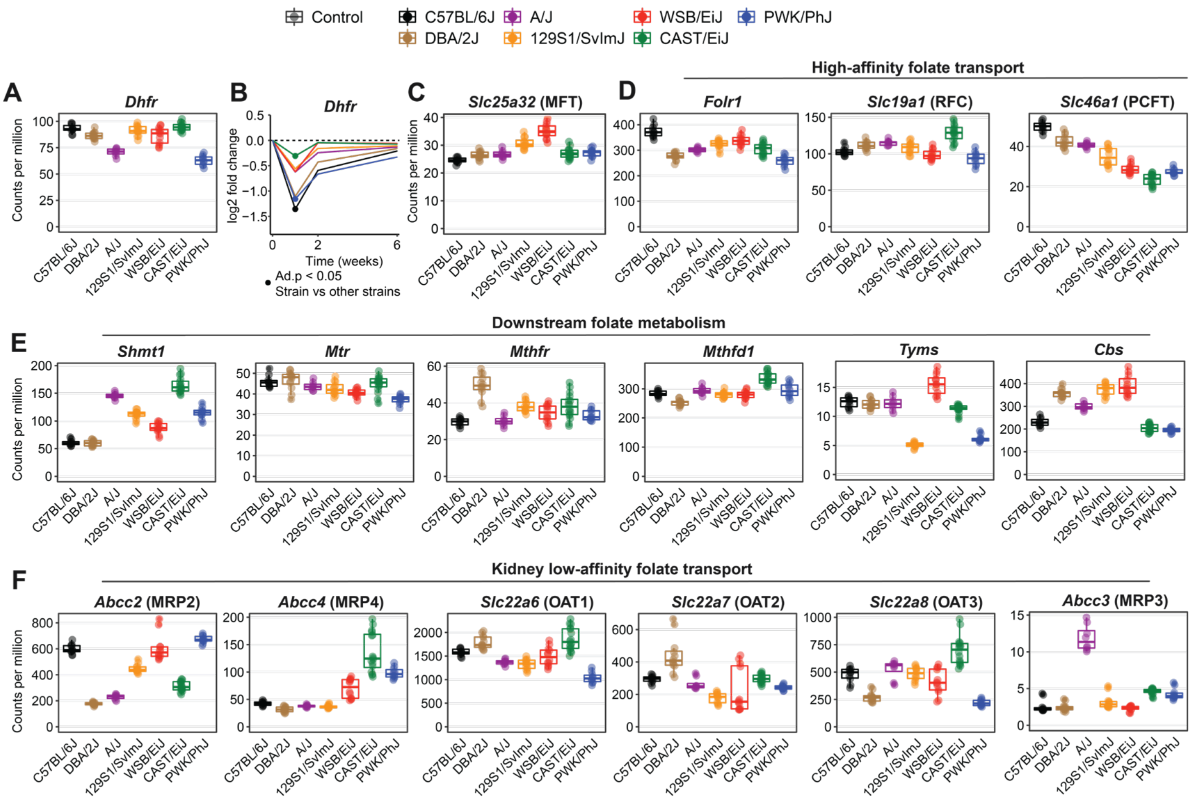

Figure S4: Expression levels of folate metabolism enzymes and transporters across strains

A: Basal expression of the folic acid metabolizing enzyme *Dhfr* across the mouse strains in control conditions (no folic acid). Expression is about 30% lower in the sensitive strain PWK/PhJ, which may contribute to its sensitivity.

B: Changes in *Dhfr* expression upon folic acid over time. The greatest loss of *Dhfr* expression is observed in the sensitive strains, but this may be a consequence, rather than a cause of the injury.

C: Basal expression of *Slc25a32*, the mitochondrial folate transporter (MFT). There are only minor changes in expression across strains, indicating that differences in mitochondrial metabolism upon folic acid may not be caused by transport of folic acid into mitochondria.

D: Expression levels of the high affinity folate transporters present in the kidney in control conditions. FOLR1 mediates glomerular transport of folate while RFC and PFCT transport into blood. While there is inter-strain variation of these transporters, it does not seem to associate with their susceptibility to injury.

E: Expression levels of folate metabolism genes acting downstream of Dhfr. While there is inter-strain variation, we do not observe consistent differences between sensitive and resistant strains.

F: Basal expression levels of the low-affinity folate transporters, such as multidrug resistance-associated proteins (MRPs) efflux pumps and organic anion transporters (OAT), which can

mediate efflux or bi-directional transport of folate. MRP2 and MRP4 mediate glomerular transport of folate while OAT 1-3 and MRP3 mediate exchanges with blood. While there is inter-strain variation of these transporters, we do not observe consistent differences between sensitive and resistant strains.

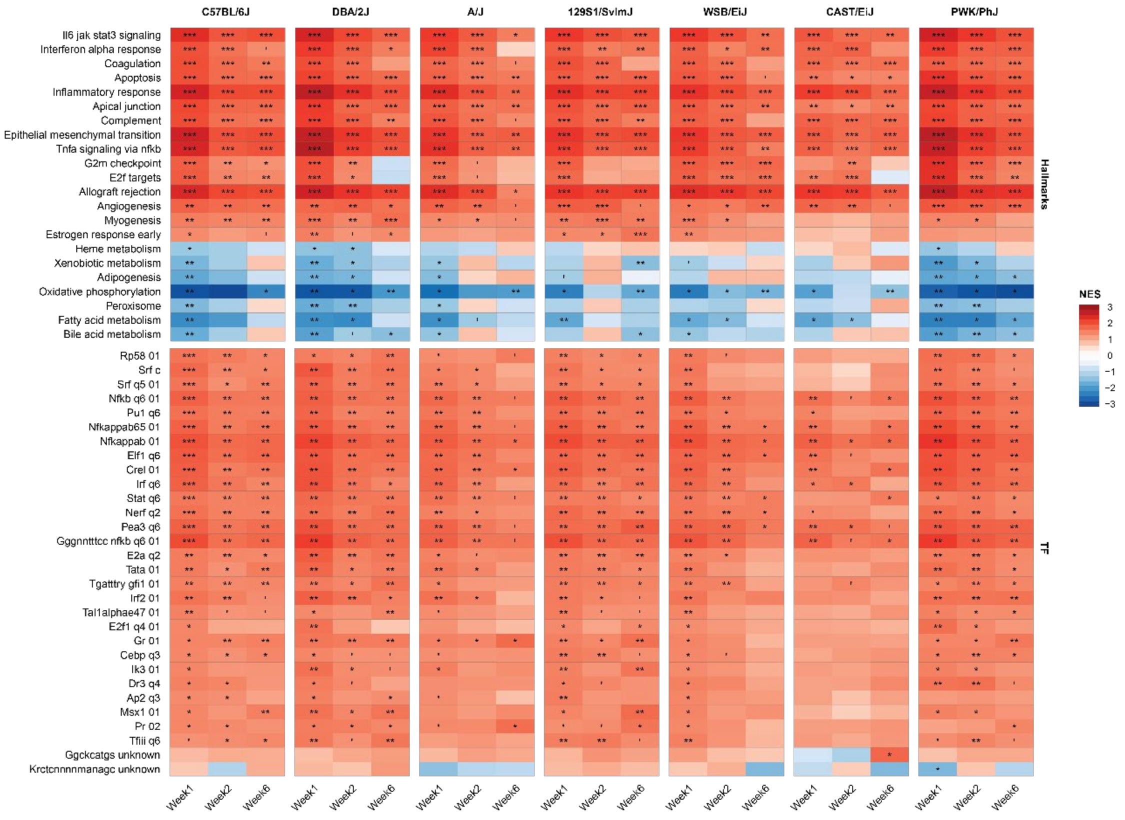

Figure S5: Extended gene set and transcription factor analysis: Gene set enrichment analysis of differentially expressed genes (treatment vs control) across strains using the top 4 gene sets enriched in each condition. Enrichments for GSEA hallmarks and transcription factors (TF) are shown.

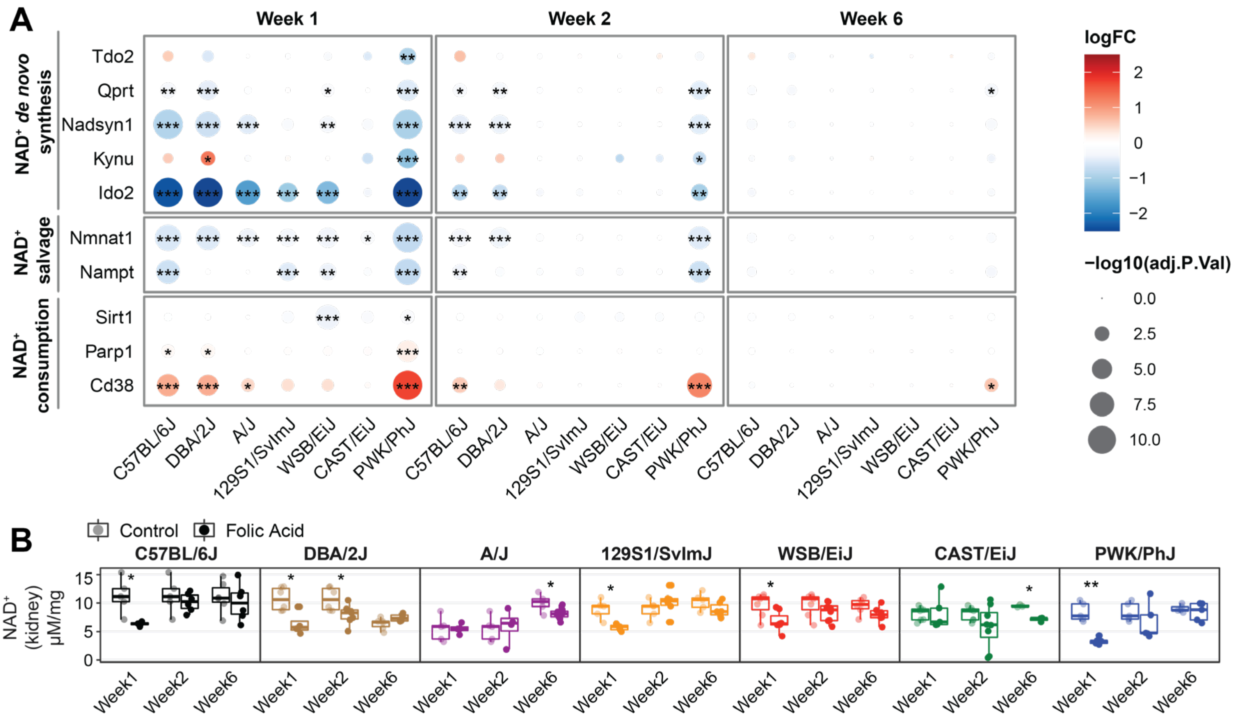

Figure S6: Alterations in the expression of genes involved in NAD⁺ homeostasis, but not in NAD⁺ itself, are retained in kidneys at week 2 after folic acid administration.

A: Effects of RNAseq-based gene expression of NAD⁺ de novo synthesis, NAD⁺ salvage and NAD⁺ consumption genes in all strains an timepoints. Colors indicate the log2 fold change and dot size denotes FDR-corrected p values ***<0.001, **<0.01, *<0.05. All strains show a general trend of downregulated NAD biosynthesis genes

B: Kidney NAD⁺ levels measured by HPLC-MS in all strains and timepoints. At weeks 2 and 6, the reduction in NAD⁺ is no longer significant in most strains.

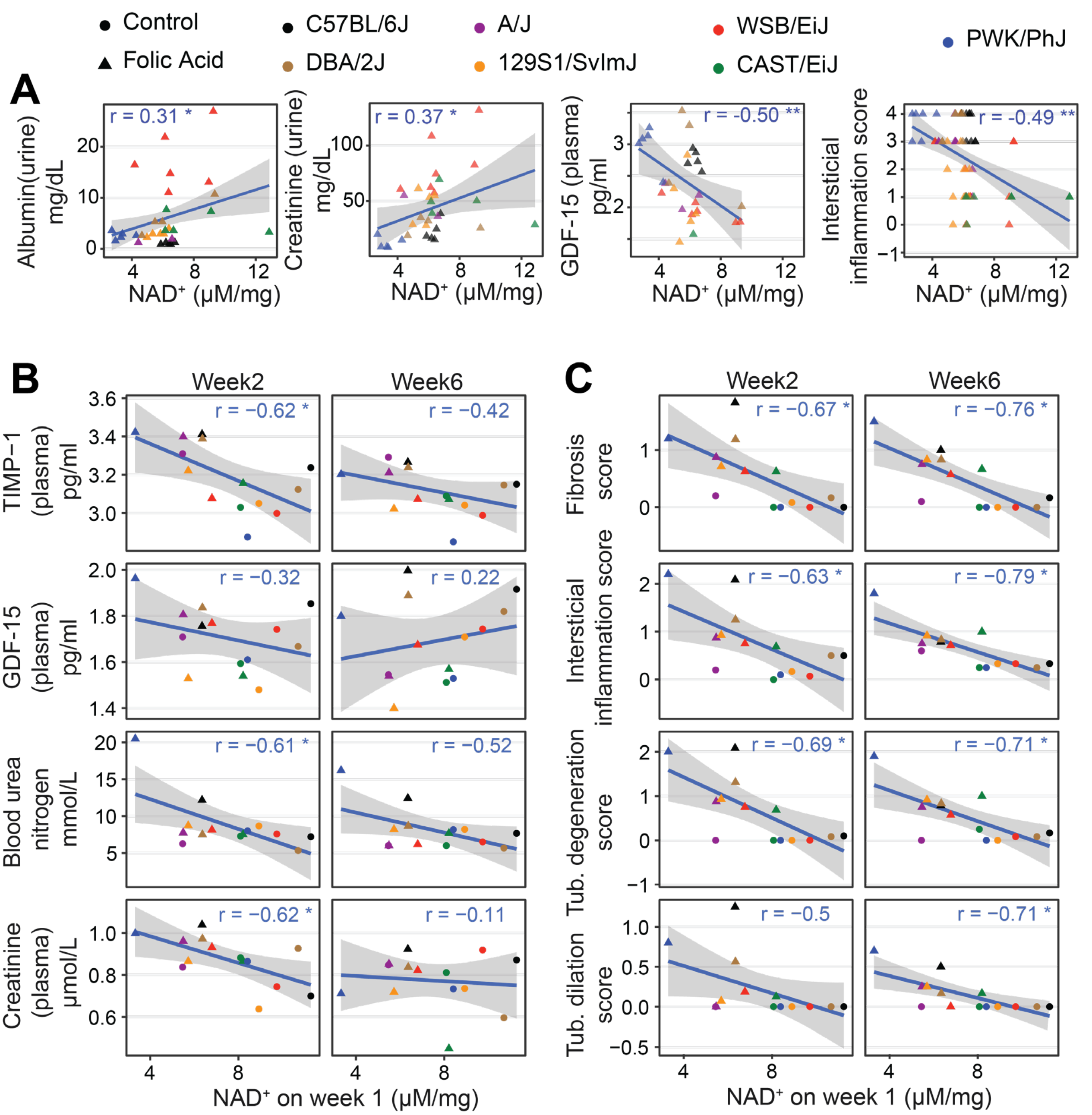

Figure S7: NAD⁺ levels correlate with disease severity at week 1, and predict later levels

A: Correlation between kidney NAD⁺ levels and additional kidney disease markers not covered on Figure 6 E-J at week 1 of folic acid administration.

B-C: Correlations between kidney NAD⁺ at week 1 and histology scores (B) and plasma parameters (C) at weeks 2 and 6. Note that unlike panel A, these correlations use averaged values per strain, to enable comparisons across different timepoints. As a result, the number of dots is reduced, but significant correlations are still retained at week 6 for all histology scores.

For all graphs, the blue line indicates a pearson linear regression. Pearson r and FDR-corrected pvalues ***<0.001, **<0.01, *<0.05, pearson correlation test, corrected for multiple testing over all possible phenotype comparisons.

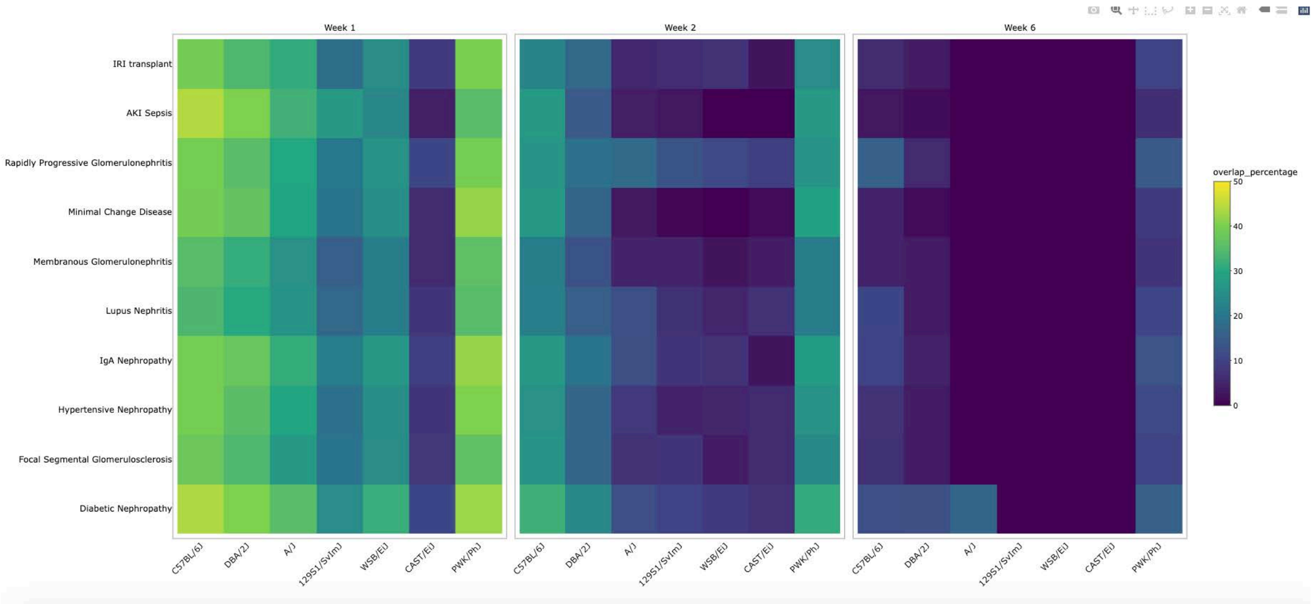

Figure S8 [html interactive graph]: Gene set over-representation analysis in overlapping DEGs across mouse and humans. Overlaps between human and mouse DEGs were selected as described in figure 7A-B, then an over-representation analysis was performed across the Biological Process (BP), cellular component (CC), KEGG pathways (KEGG) and GSEA hallmarks (Hallmarks) databases downloaded from mSigDB v7.5.1. The top 5 pathways at FDR-corrected pvalue <0.05 are indicated in the interactive tooltips that display for each overlap. An overview of the most significant gene sets found in this manner is represented on Figure 7C-D.

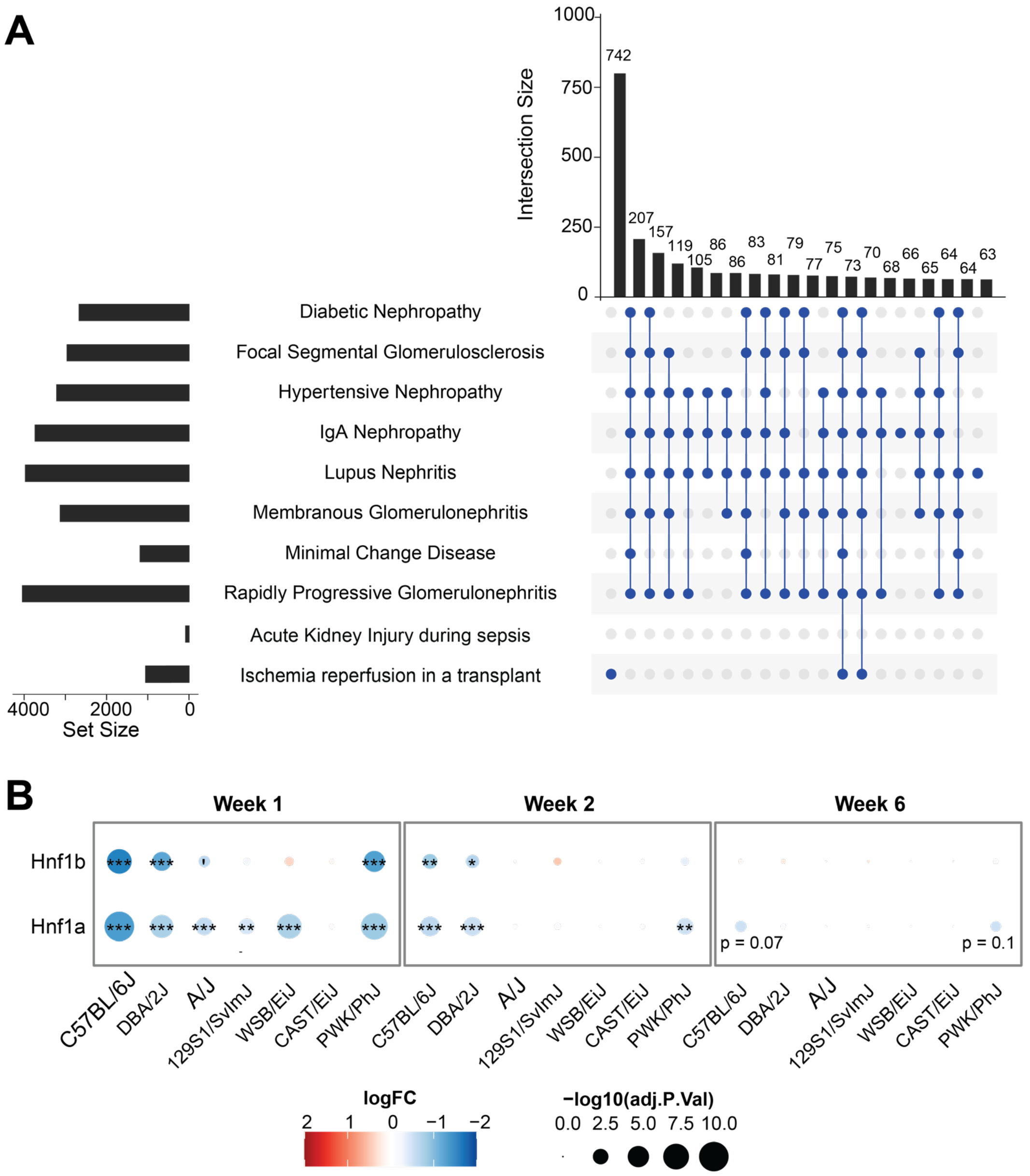

Figure S9: Human disease overlaps and *Hnf1* expression.

A: Upset plot of the overlaps between human DEGs across the diseases used for comparison in Figure 7. AKI and IRI share very few DEGs with CKD-related disease, with most of the AKI-related genes being unique. 207 transcripts are shared between all CKD-related diseases and the rest of the genes are shared between smaller subsets of diseases.

B: Fold change of the expression of *Hnf1a* and *Hnf1b* across all mouse strains and conditions. The C57BL6/J and PWK/PhJ strains behave in a similar manner, with an early downregulation of both genes, which is lost over time, and an almost-significant downregulation of *Hnf1a* at week 6.

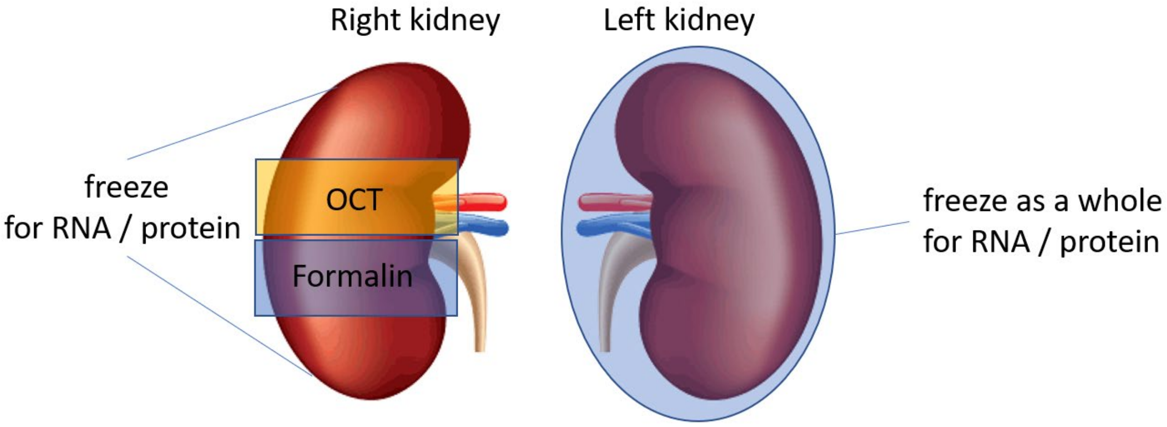

Figure S10: Kidney splitting protocol for RNA/protein isolation and histology.

Stress response	Selected genes	Source	Selection method
Mitochondrial SR	Common signature	1	The signature of the UPRmt reponse: genes that were upregulated upon every different source of mitochondrial stress
ER SR	XBP1 targets	2	Targets that had Chipseq binding site for XBP1, and were upregulated upon IRE1a inhibition (FC < -0.5 only)
Integrated SR	Atf4 & Chop targets	3	Table S5 from the paper: genes that were directly regulated by ATF4 and CHOP (Chip_seq and induced by ISR): only fold changes >0
Heat shock response	Hsf1 targets	4	Table S4 from the paper: HSF1 target genes in mouse by tissue/cell type. 'Shared targets': List of HSF1 target genes shared between at least two examined cell types in mouse, only direct interaction
Oxidative SR	Nrf2 targets	5	Table S4 from the paper: genes with Chip-seq binding site and part of the Nrf2 inducible response
ISGs	Type I IFN response	6	Table S2: Mouse ISGs

Table S1: References and selection method of the stress response genes used in Figure 5B. The gene sets were selected to be as "core" as possible. For transcription factors only genes that had Chipseq-confirmed binding and were upregulated after activation are included. For the MSR, only overlapped genes for all three stimulations are included.

Disease	GEO databaset	Source
Ischemia reperfusion in a transplant	GSE30718	7
Acute kidney injury during sepsis	GSE139061	8
CKD signatures	GSE20602, GSE32591, GSE37460, GSE47183, GSE50469	9

Table S2: References to the public repositories for the used human data

References for tables S1 and S2:

1. Quirós, P. M. *et al.* Multi-omics analysis identifies ATF4 as a key regulator of the mitochondrial stress response in mammals. *J. Cell Biol.* **216**, 2027–2045 (2017).
2. Pramanik, J. *et al.* Genome-wide analyses reveal the IRE1a-XBP1 pathway promotes T helper cell differentiation by resolving secretory stress and accelerating proliferation. *Genome Med.* **10**, 76 (2018).
3. Han, J. *et al.* ER-stress-induced transcriptional regulation increases protein synthesis leading to cell death. *Nat. Cell Biol.* **15**, 481–490 (2013).
4. Kovács, D. *et al.* HSF1Base: A Comprehensive Database of HSF1 (Heat Shock Factor 1) Target Genes. *Int. J. Mol. Sci.* **20**, 5815 (2019).
5. Malhotra, D. *et al.* Global mapping of binding sites for Nrf2 identifies novel targets in cell survival response through ChIP-Seq profiling and network analysis. *Nucleic Acids Res.* **38**, 5718–5734 (2010).
6. Wu, X. *et al.* Intrinsic Immunity Shapes Viral Resistance of Stem Cells. *Cell* **172**, 423-438.e25 (2018).
7. Tajti, F. *et al.* A Functional Landscape of CKD Entities From Public Transcriptomic Data. *Kidney Int. Rep.* **5**, 211–224 (2020).
8. Janosevic, D. *et al.* The orchestrated cellular and molecular responses of the kidney to endotoxin define a precise sepsis timeline. *eLife* **10**, e62270.
9. Park, M., Kwon, C. H., Ha, H. K., Han, M. & Song, S. H. RNA-Seq identifies condition-specific biological signatures of ischemia-reperfusion injury in the human kidney. *BMC Nephrol.* **21**, 398 (2020).
